# A Comprehensive 2018-Based Vehicle Emission Inventory and Its Spatial–Temporal Characteristics in the Central Liaoning Urban Agglomeration, China

**DOI:** 10.3390/ijerph19042033

**Published:** 2022-02-11

**Authors:** Yingying Liu, Xueyan Zhao, Jing Wang, Shengnan Zhu, Bin Han, Di Zhao, Xinhua Wang, Chunmei Geng

**Affiliations:** 1 State Key Laboratory of Environmental Criteria and Risk Assessment, Chinese Research Academy of Environmental Sciences, Beijing 100012, China; liuyy@craes.org.cn (Y.L.); zhaoxy@craes.org.cn (X.Z.); wangjing@craes.org.cn (J.W.); hanbin@craes.org.cn (B.H.); 2 Shenyang Academy of Environmental Sciences, Shenyang 110167, China; zhushengnan@syhky.com (S.Z.); zhaodi@syhky.com (D.Z.)

**Keywords:** vehicle emissions, emission standard, Central Liaoning Urban Agglomeration

## Abstract

Rapid economic expansion and urbanisation have seriously affected the atmospheric environmental quality of the Central Liaoning Urban Agglomeration (CLUA). This study aimed to establish a detailed vehicle emission inventory of the CLUA with a 3 km × 3 km gridded spatiotemporal distribution. A top-down methodology using vehicle kilometres travelled annually, emission factors, and activity data of each city was established. Carbon monoxide (CO), nitrogen oxides (NOx), sulfur dioxide (SO_2_), ammonia (NH_3_), volatile organic compounds (VOCs), particulate matter with an aerodynamic diameter less than 2.5 μm (PM_2.5_), particulate matter with an aerodynamic diameter less than 10 μm (PM_10_), Black Carbon (BC), and organic carbon (OC) emissions were 291.0, 221.8, 3.6, 2.2, 42.8, 9.3, 10.3, 5.2, and 1.6 Gg in 2018, respectively. The contribution of diesel heavy-duty trucks to NOx, SO_2_, PM_2.5_, PM_10_, BC, and OC emissions was greater than 54.5%, the largest contribution of all vehicles. Gasoline small passenger vehicles were the primary contributor to CO, VOC, and NH_3_ emissions, contributing 37.3%, 39.5%, and 75.3% of total emissions, respectively. For emission standards, Pre-China 1 vehicles were the largest contributor to CO and VOC emissions and China 3 vehicles contributed the largest amount of NOx, SO_2_, PM_2.5_, PM_10_, BC, and OC emissions. The spatial distribution of pollutants showed “obvious lines” and grids with high emissions were concentrated in expressways, national highways, and provincial highways. The temporal variation showed morning–evening peaks during diurnal variations, which was consistent with resident behaviour. This work can help us understand vehicular emission characteristics of the CLUA and provide basic data for air quality modelling. Future research should investigate traffic flow by vehicle types and emission factors at a local level, which will be helpful for transport management planning.

## 1. Introduction

With rapid economic development and urbanisation during the past four decades in China, vehicle ownership and population have increased sharply from 1.35 million in 1978 to 232.31 million in 2018 [1]. Vehicles contribute significantly to air pollutant emissions in China’s megacities and are a vital cause of fine particulate matter and photochemical smog pollution [2]. In particular, transportation emissions accounted for 6.7% of greenhouse gas (GHG) emissions in China in 2014 [2]. Moreover, the national total GHG emissions are expected to continue to grow as vehicle ownership increases. Meanwhile, pollutants from vehicle emissions will directly endanger public health [3,4].

Comprehensive vehicular emission inventories can quantify the air pollutants emitted from vehicles [5] and are crucial for the management of air pollution and to assist in policy-making. Given the vital impact of vehicles on pollutants and GHG emissions in China, it is of great importance to estimate vehicle emissions with high spatiotemporal resolution. Vehicle emissions have been estimated at the national [6], provincial [7], and city [8] levels in many studies in China. A review of typical cities that established vehicle emission inventories with different methods is summarised in Table S1.

As shown in Table S1 , the International Vehicle Emissions (IVE) model, Computer Program to calculate Emissions from Road Transport (COPERT) model, MOBILE, MOVES, and Chinese guides are widely used to determine the vehicle emission inventory in China. Other methods, including portable emission measurement systems (PEMS) [9,10] and on-road remote sensing measurements [11], have been widely used to monitor real-world on-road vehicle emissions. Several researchers have developed new methods or optimised existing methods to establish vehicle emission inventories [12,13]. Researchers have attempted to improve the accuracy of those cities and regions that already have an established vehicle emission inventory. 

Liaoning province, which is the most important industrial base in northeast China, suffers from significant air pollution during cold winters. Vehicle fuel consumption is an important emission source of PM_2.5_ [14]. The spatial and temporal distribution of SO_2_, NO_2_, and PM_2.5_ shows a general trend of high emissions occurring in the central part of Liaoning province compared to the eastern and western regions [15]. Vehicle exhaust emissions contributed 7–13% to annual PM_2.5_ pollution and 30–55% to NO_2_ in 14 cities of Liaoning in 2016 [15]. For Shenyang city, vehicle emissions contributed 55% to NO_2_ in 2016 and 37.4% to polycyclic aromatic hydrocarbons (PAHs) in PM_2.5_ during 2014–2018 [16]. The implementation of Liaoning’s economic revitalisation plan has involved considerable industrial development, resulting in a corresponding increase in exhaust pollution due to freight vehicles [17], as economic development has led to an increase in the number of transport vehicles [18]. Therefore, the impact of motor vehicle emissions on air pollution in Liaoning should attract more attention, especially in the Central Liaoning Urban Agglomeration (CLUA) region.

Anthropogenic emission inventories of air pollutants can provide foundational data for air quality warning and forecast platforms. Vehicle emission inventories are critical for supporting the strategic approach for managing transport emissions that has been adopted by the local government in the CLUA. Previous studies have examined Shenyang [19], Benxi [20], Liaoning Province [21,22], and NEC [23]. However, these are only a small component of the CLUA, and the studies were conducted over different years, with different methods, pollutants, or lack of time and space resolution. Thus, these studies are insufficient to properly advise local air quality warning and forecast systems. The purpose of the current study is to establish a comprehensive regional and high-resolution vehicle emission inventory in the CLUA region.

The present study established a basic vehicle emission inventory for the CLUA region using a top-down methodology and distributed it in a 3 km × 3 km grid. Pollutants studied include O_3_ precursors, aerosol precursors, and aerosols (CO, NOx, SO_2_, NH_3_, VOCs, PM_2.5_, PM_10_, BC, and OC). The methods of emission inventory, spatial allocation, and uncertainty analysis are clarified in Section 2. The traffic composition, emission characteristics, spatial distribution of emissions, and daily and weekly variations are discussed in Section 3. The establishment of emission inventory is complicated, although lots of assumptions are there in the emission inventory. Any emission inventory can lead to the facilitation of planning strategies and can be rectified further. In this work basic emission factors and traffic flow are cited literatures and have been corrected with the actual situation in CLUA. Evaluating hypothetical assessment is very important and the assessment of uncertainties is presented in this work. However, there are still many limitations, such as no measured EFs and the lack of traffic flow data by vehicle type. Future work will continue to refine these vehicle emissions data. 

## 2. Materials and Methods

### 2.1. Study Area

**Figure 1:**
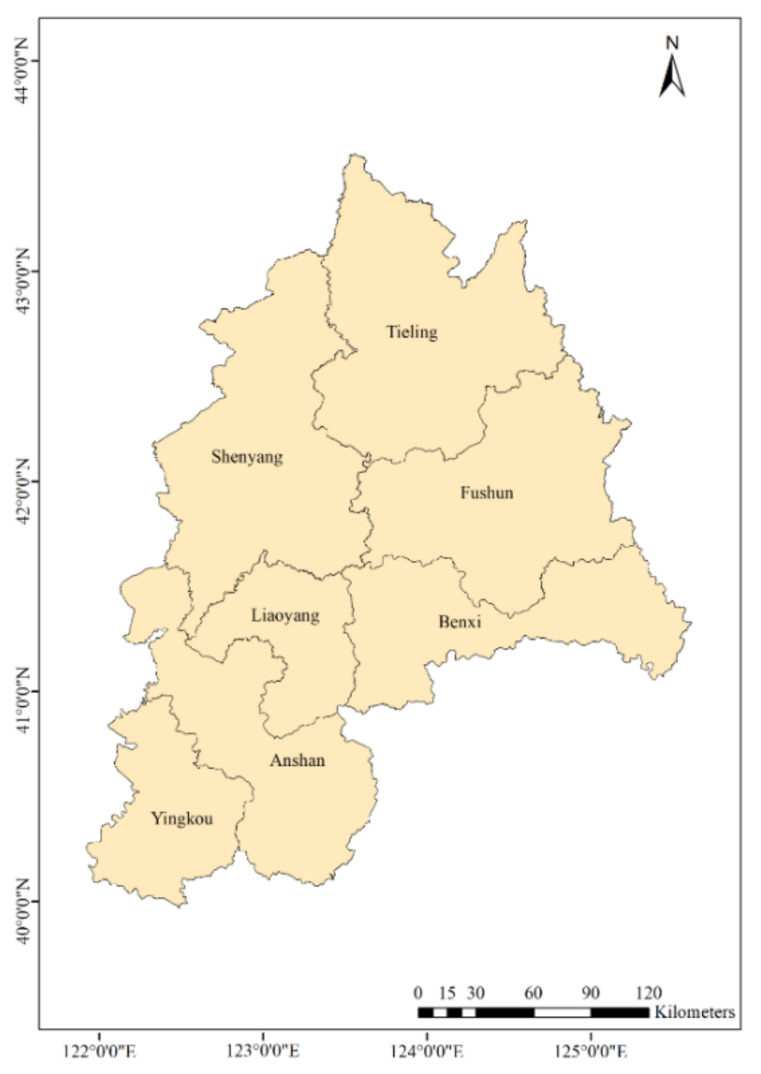
The Central Liaoning Urban Agglomeration (CLUA) and its location.

The CLUA region is located in central Liaoning Province, as shown in Figure 1. Shenyang is on the west of the CLUA, surrounded by five cities: Anshan, Benxi, Fushun, Liaoyang, and Tieling. Yingkou is located on the east coast of the Bohai Sea. The CLUA covers an area of 65,000 square kilometres, accounting for 44% of Liaoning Province. At the end of 2019, the total population of the CLUA was estimated at 21.4 million, which was 51.1% of the total population in Liaoning [24]. The CLUA accounts for 50.8% of the total national gross domestic product (GDP) of Liaoning Province and the local fiscal revenue accounted for 54.2% of the total in 2019 [24]. The civilian vehicle population was 4.7 million, an increase of 20.1% from 2016 to 2019 in CLUA. At the same time, private car ownership was 4.1 million, an increase of 43.1% [24,25]. 

### 2.2. Emission Inventory Methodology

#### 2.2.1. Investigation and Collection of Vehicle Activity Level Data

Data on the activity levels of mobile road sources had to be investigated, including the number of different types, oil products, and emission standards of on-road vehicles in each city. The data sources used for the establishment of the CLUA vehicle inventory are briefly summarised in Table 1. All cities had detailed information on the vehicle types, fuel type, and emission standards, except for Tieling. The vehicle quantity of Tieling was similar to that of Liaoyang; therefore, the vehicle type, fuel type, and emission standards were allocated based on the Liaoyang proportions.

**Figure 2:**
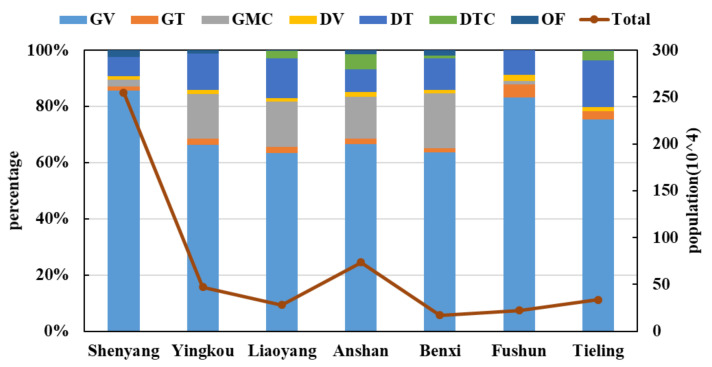
Total vehicle quantities and distribution by type in the CLUA region during 2018.

The distribution of vehicle ownership for different fuel types in various cities in the CLUA is shown in Figure 2. According to the technical guidelines on emission inventory (GEI) released by the Ministry of Ecology and Environment of the People’s Republic of China (MEE) [26], the categories were: gasoline passenger vehicles (GV), gasoline trucks (GT), gasoline motorcycles (GMC), diesel passenger vehicles (DV), diesel trucks (DT), diesel tricycles (DTC), and other fuel (OF) vehicles. OF vehicles mainly included natural gas vehicles, liquefied petroleum gas vehicles, and hybrid electric vehicles. Gasoline vehicles were the main vehicles in the CLUA region, accounting for 78.2–89.5% of all vehicles, with diesel vehicles accounting for 8.2–21.5%, and OF vehicles accounting for 0–2.4%. The gasoline vehicles were mainly passenger vehicles, whereas the diesel vehicles were mainly freight vehicles.

#### 2.2.2. Emission Estimations

The on-road vehicle emissions were calculated by EF and activity data at the city level. In the present study, the vehicle emission inventories of CO, NOx, SO_2_, NH_3_, VOCs, PM_2.5_, PM_10_, BC, and OC were calculated using the following Equation (1) [7,27,28,29,30], which was fit for evaporative emissions and operational emissions:

(1) $$E_{ij}=\underset{j}{\sum }\underset{i}{\sum }(P_{ij}\times EF_{ij}\times VKT_{ij})\times 10^{-6}$$

where *E* is the annual emissions (t/a) of CO, NOx, SO_2_, NH_3_, VOCs, PM_2.5_, PM_10_, BC, and OC; *P* is vehicle population; *EF* is the emission factor, in grams of pollutant per unit distance for each single vehicle in g/km; *VKT* is the annual vehicle kilometres travelled, obtained from the GEI [26]; and j represents the national vehicular emission limit standard (Pre-China 1, China 1, China 2, China 2, China 3, China 4, and China 5 emission standards). i is the vehicle type, including 12 diesel vehicles (heavy-duty DTs, medium-duty DTs, light-duty DTs, mini diesel trucks (MDTs), DTCs, low-speed DTs, heavy-duty DVs, medium-duty DVs, medium-duty passenger vehicles (MDPV), small DVs, diesel taxis, and diesel buses), 12 gasoline vehicles (heavy-duty GTs, medium-duty GTs, light-duty GTs, mini GTs, heavy-duty GVs, medium-duty GVs, small GVs, mini GVs, gasoline taxis, gasoline buses, normal gasoline motorcycles, and light gasoline motorcycles), and nine OF type vehicles, (OF buses, OF taxis, OF HDTs, OF medium-duty trucks, OF light-duty trucks, OF heavy-duty vehicles, OF medium-duty vehicles, OF small passenger vehicles, and OF mini passenger vehicles). As known to all, other vehicle specifications such as engine capacities have an important effect on real-world emissions. Information on the engine capacities of the vehicles from activity level data was not collected in this work. We will investigate and make up for the information in future research.

#### 2.2.3. Calculation of Emission Factors

On-road vehicle EFs were simulated, taking into account the local conditions. The method was described in the GEI, and involved the following equation [26]:

(2) EF=BEF×ϕ×γ×λ×θ

(3) $$\phi =\phi_{Temp}\times \phi_{RH}\times \phi_{Height}$$

where the basic emission factor (*BEF*) is the base *EF* (g/km), displayed in Table S2; BEFs were obtained from the technical guidelines on emission inventory (GEI) released by the Ministry of Ecology and Environment of the People’s Republic of China (MEE) [26]. These BEFs were tested by researchers of Tsinghua University and details were described in previous studies [31,32,33,34]. *ϕ* represents the meteorological correction parameters (temperature, humidity, and height in each city) (shown in Table S3). γ refers to the average speed correction coefficient, λ is the degradation correction coefficient, and θ refers to other correction coefficients, calculated as the product of the sulfur content correction coefficient, load coefficient, and oil quality. Detailed information for *ϕ*, γ, λ, and θ is displayed in Tables S4, S8–S12. The fuel quality information (Table S4) was obtained from the Shenyang Environmental Protection Bureau. The diesel vehicle load ratio was assumed as 100% loaded. The gasoline and OF vehicle load ratio were taken as the default value from the GEI. 

#### 2.2.4. Spatial Distribution 

In the present study, a spatial grid of 3 km × 3 km resolution for the vehicle emission inventory containing 7380 grids was obtained using ArcGIS software (as shown in Figure S4). The spatial distribution of vehicle emissions used the road-network-based approach, which has been described in previous studies [8,35,36] and showed more reasonable variations due to the changes in road types and traffic flow. The spatial distribution of vehicle emissions considered vehicle travel pattern (the usual type of vehicle on each road type) and volume (number of vehicles on each road type at the same time). The spatial allocation of vehicle emissions was processed as follows. Firstly, the traffic flow of different road types of each city was used to calculate the distribution coefficients of different vehicles for each road type (named *A*). Then, the emissions of each vehicle type on each road type were acquired by multiplying *A* with the emission inventory of each vehicle type (named *B*). Thus, the sum of the emissions of *B* for each road type was the emission inventory for that road type for each city (named *EIERY*). Secondly, the total road length of each road type for each city of the CLUA was known (named *C*). The road length of each road type in each 3 km × 3 km grid was obtained from the GIS (named *D*). Thus, distribution coefficients for different road types within each grid were calculated by dividing *D* by *C*. The emissions for each road type within each grid were obtained by multiplying *EIERY* by the coefficients of each road type within each grid. The sum of the emissions for all road types within each grid for different pollutants was the emission inventory of each grid. Finally, the emission inventory of the CLUA was distributed into 7380 grids at 3 km × 3 km spatial resolution. The formula for the emissions of each grid was as follows [8,37]:

(4) $$E_{n,j}=\underset{i}{\sum }\underset{c}{\sum }\frac{L_{n,c,i}}{Li,c}\times E_{i,c,j}$$

where *n* and *j* are the grid number and type of pollutant, respectively; *c*, *i*, and *L* represent the road types, region, and road length of different types, respectively; and *E_n,j_* refers to the emissions of pollutant *j* in grid number *n*.

In the present study, there were nine different road types in the CLUA region. Urban roads were divided into urban expressways, main roads, secondary roads, and branch roads, and roads outside the urban area included expressways, national highways, provincial highways, county roads, and rural roads (Figure S1). The daily traffic flows for the vehicle fleet were acquired from previous studies on Harbin, which is located in northeast China [36]. A spatial distribution map of traffic flow is shown in Figure S5.

#### 2.2.5. Uncertainty Analysis

The uncertainty of the vehicle emission inventory was measured using the uncertainties and variability in the emission factors and activity data. Road traffic emissions were the main sources of uncertainty in the air quality numerical models. Therefore, the accuracy of the quantitative evaluation list was important.

In the present study, the uncertainties in the emission factors and activity level data were taken into consideration when determining the uncertainty of the inventory. For the data on activity levels, the population of each vehicle category and the vehicle kilometres travelled (VKT) were obtained by questionnaire (as discussed in sub-Section 2.2.1). Where VKT was not provided in the questionnaire, values were sourced from the GEI [26], which may contribute to uncertainty in calculating vehicle sources. The Monte Carlo method was used to quantify the uncertainties of the various pollutants in the CLUA region, as used in previous studies [10,12,38,39]. Average values and ranges were used to quantify the uncertainties of the emission inventory.

According to each fuel type-vehicle type combination, the uncertainty of the emissions of various pollutants for different vehicle types under different fuel types was calculated. The premise was that the uncertainties in the activity data and emission factors could be described by a normal distribution. The trial number was set to 10,000 and the confidence level was set to 95%.

## 3. Results and Discussion

### 3.1. Traffic Composition

Based on the activity level data of the CLUA region, vehicles were divided according to fuel and vehicle types and emission standards (Table 2). Gasoline vehicles accounted for 86.6% of the total number of vehicles in the CLUA region, of which SPVs were the main type, accounting for 87.2%. Diesel vehicles comprised only 11.7% of total vehicles in the CLUA region. Among these, HDT and light-duty trucks (LDT) accounted for a relatively high proportion (33.3% and 30.7%, respectively). Vehicles of OF types had the lowest proportion. For the emission standards, China 4 models had the largest number of vehicles, accounting for 45.1%, followed by China 3 and China 2, which accounted for 24.9% and 11.1% of the total number of vehicles in the CLUA region, respectively.

### 3.2. Vehicle Emission Inventory Characteristics

#### 3.2.1. Emission Characteristics by Vehicle Type and Fuel Type

**Figure 3:**
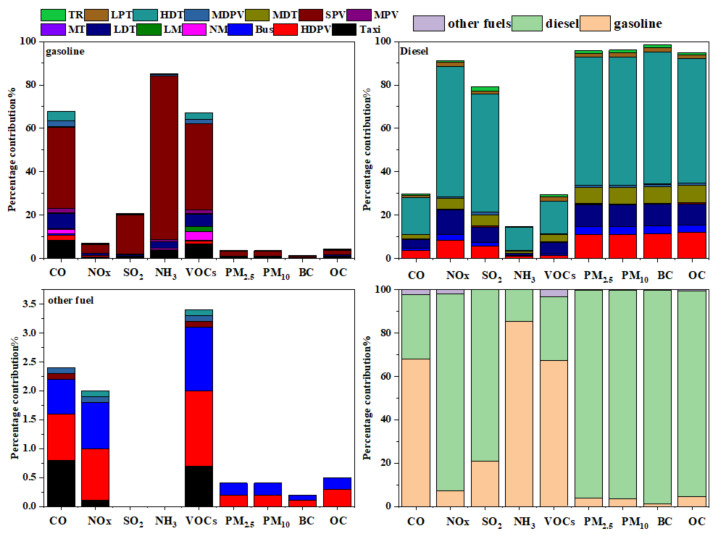
Distribution of multi-pollutant emissions of different vehicle types in 2018 in the CLUA.

In 2018, vehicles emitted 291.0 Gg of CO, 221.8 Gg of NOx, 3.6 Gg of SO_2_, 2.2 Gg of NH_3_, 42.8 Gg of VOCs, 9.3 Gg of PM_2.5_, 10.3 Gg of PM_10_, 5.2 Gg of BC, and 1.6 Gg of OC in the CLUA region. The different pollutants and their contribution by different vehicle and fuel types are summarised in Figure 3 and Table S5.

Diesel HDTs contributed significantly to NOx, SO_2_, PM_2.5_, PM_10_, BC, and OC emissions in the CLUA region (59.9%, 54.5%, 58.8%, 59.2%, 61.1%, and 57.2%, respectively). This was mainly due to overloading and low driving speed leading to insufficient diesel fuel burning, causing an increase in NOx and particulate emissions [40]. Diesel LDTs were the second-highest contributor to NOx and gasoline SPVs were the second-highest contributor to SO_2_ at 11.3% and 18.1%, respectively. Diesel HDPVs were the second-largest contributor to PM_2.5_, PM_10_, BC, and OC emissions, higher than that of diesel LDTs. Diesel HDTs contributed 16.8%, 10.6%, and 14.9% to CO, NH_3_, and VOC emissions, respectively. Diesel HDPVs, accounting for 0.5% of the total vehicle ownership in the CLUA region, were the second-largest contributor to PM_2.5_, PM_10_, BC, and OC emissions, responsible for 11.1%, 11.1%, 11.3%, and 11.8%, respectively. Gasoline SPVs were the primary contributor to CO, VOC, and NH_3_ emissions, contributing 37.3%, 39.5%, and 75.3% of total emissions, respectively.

For NOx, SO_2_, PM_2.5_, PM_10_, BC, and OC emissions, diesel vehicle fleets were the major contributors among all vehicle categories, especially HDTs, whereas CO, NH_3_, and VOC emissions were mainly due to gasoline SPVs. Vehicle emission characteristics in the CLUA region are consistent with previous studies in Guanzhong [41], Henan Province [36], and China [42]. 

Based on these findings, restricting the use of small passenger vehicles is critical to reducing CO, VOC, and NH_3_ emissions. In addition, improvement of diesel quality should be urgently considered regarding NOx, SO_2,_ and PM emissions. In terms of fuel types (Figure 3 and Table S5), diesel vehicles were the primary contributors to NOx, SO_2_, and PM, with rates between 79.2% and 98.5%. Gasoline vehicles were the main emission sources of CO, NH_3_, and VOCs, accounting for 67.9%, 85.4%, and 67.2%, respectively. Compared with diesel and gasoline vehicles, OF vehicles, which only accounted for 1.7% of total ownership, were more environmentally friendly. Therefore, improving the proportion of vehicles powered by non-fossil fuels, including public transportation, and limiting the number of private vehicles, are also important measures to reduce pollutant emissions.

#### 3.2.2. Emission Characteristics of Sub-Emission Standards 

**Figure 4:**
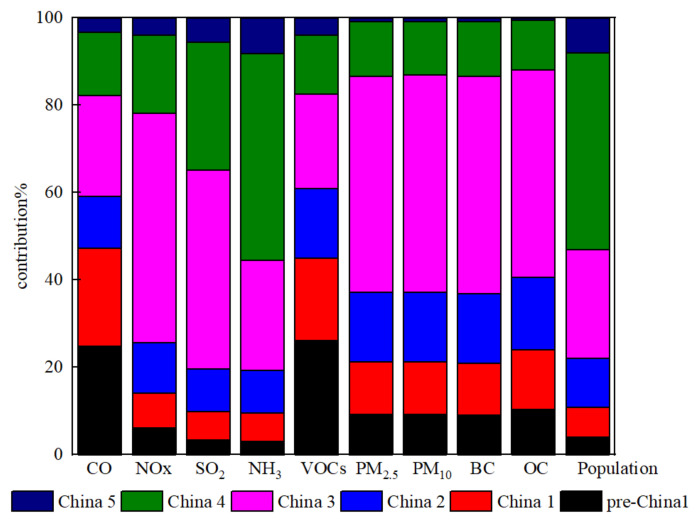
Emission contributions of vehicles with different emission standards.

According to the data obtained from the survey, the emission standards for vehicle sources in the CLUA region included Pre-China 1, China 1, China 2, China 3, China 4, and China 5. Figure 4 shows the proportion of vehicle ownership with different emission standards and the share of pollutant emissions in the CLUA region. The contribution of vehicles with different emission standards to pollutant emissions varied greatly. In terms of vehicle quantities, the proportion of China 4 was the largest, up to 45.1% of the total vehicles, followed by China 3 and China 2, accounting for 24.9% and 11.1%, respectively. Pre-China 1, China 1, and China 5 vehicles accounted for a relatively low proportion at 4.1–8.0%. Although vehicles pre-dating China 3 accounted for only a small proportion, they contributed 59.1% of total CO and 61.0% of total VOCs. Pre-China 1 vehicles had the highest CO and VOC emissions, with rates of 24.8% and 26.1%, respectively, approximately six times the proportion of vehicle quantity. China 3 vehicles had the largest share of NOx, SO_2_, PM_2.5_, PM_10_, BC, and OC emissions, ranging from 45.5% to 52.6%, approximately twice the proportion of their ownership. The rate of NH_3_ emissions by vehicles of various emission standards was equivalent to that of the vehicle quantities, and China 4 vehicles contributed the greatest amount of NH_3_ emissions. In contrast, these vehicles had relatively small contributions to CO, NOx, SO_2_, VOCs, PM_2.5_, PM_10_, BC, and OC emissions, accounting for 12.0–29.2%, which was much lower than their vehicle quantity.

Among the nine pollutants emitted by mobile road sources, CO, NOx, and VOC emissions were significantly higher than the other six pollutants. Pre-China 1 and China 1 vehicles, which accounted for 10.9% of the total vehicle fleet, contributed 47.4% of CO and 45.0% of VOC emissions. Pre-China 1 and China 1 vehicles remained the main contributors to CO and VOC emissions. China 3 and China 4 vehicles were the major contributors to NOx, SO_2,_ and PM emissions (Figure 3) because 79.3% of the HDTs operated under China 3 and China 4 emission standards. Previous research has shown that eliminating high-emission old cars in the short term is an effective measure to reduce emissions [43]. Therefore, it is recommended that governments in the CLUA region eliminate old vehicles and implement strict emission standards for motor vehicles.

#### 3.2.3. Characteristics of Each City

The vehicle emissions in each city in the CLUA region are shown in Table 3. Significant differences were observed in the nine pollutants among the seven cities, with the highest vehicle emissions in Shenyang (218.0 kilometric tonnes (kt)), followed by Anshan city (148.2 kt). Vehicle emissions in Yingkou were comparable to those in Tieling, and emissions in Fushun were comparable to those in Liaoyang. Benxi had the lowest emissions, accounting for 3.2% of total emissions. The reasons for these differences in emissions between cities were related to the number of mobile road sources in each city, and the fuel type, vehicle model, and emission standard distributions of the mobile road sources. Shenyang is the capital of Liaoning Province, and had the highest quantities of SPVs, accounting for 45.9% of the total CLUA region (Figure S2b). CO, NOx, SO_2_, NH_3_, VOCs, PM_2.5_, PM_10_, BC, and OC emissions in Shenyang contributed 39.3%, 33.4%, 36.2%, 51.6%, 41.7%, 35.3%, 35.2%, 34.8%, and 34.8% of total emissions in the CLUA, respectively. Anshan had the second-highest rate of vehicle ownership in the CLUA region, and the number of SPVs ranked second among the seven cities, accounting for 10.0%. Emissions of the nine pollutants in Anshan accounted for 15.5–26.5%, which was second only to that of Shenyang.

**Figure 5:**
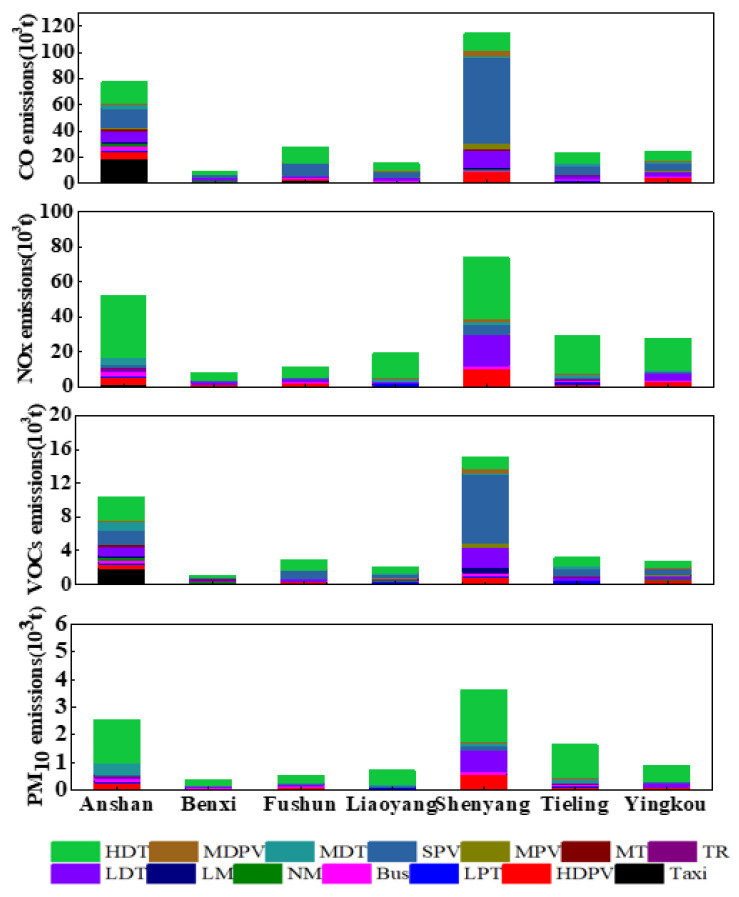
Emission intensity of different vehicle types in each city during 2018.

To further investigate the emissions of pollutants in the different cities and provide decision-makers with relevant vehicle management information, the emission characteristics of the different cities were considered. Figure S3 depicts the emission contribution of the different vehicle types in the CLUA region to CO, NOx, VOCs, and PM_10_ in 2018. SPVs contributed the largest amount to CO and VOC emissions, due to their large quantities, followed by HDTs (Figure S2a). HDTs were the primary contributor to NOx and PM_10_. In the present study, we selected CO, NOx, VOCs, and PM_10_, which were the top four pollutants with high emissions, to analyse the emission in each city in the CLUA region. The emissions from each vehicle type in each city are shown in Figure 5. In Shenyang, SPVs contributed 57.4% (65.7 kt) and 54.7% (8.3 kt) of CO and VOC emissions, respectively. The second-largest contributor was LDTs, contributing 12.1% (13.8 kt) of CO and 15.6% (2.4 kt) of VOCs. In Anshan, taxis, HDTs, and SPVs were the three main vehicle types contributing to CO and VOC emissions. In Fushun, Liaoyang, Tieling, and Yingkou, SPVs and DTs were the main vehicle types contributing to CO and VOC emissions.

For NOx emissions in Shenyang, HDTs were the primary contributor, accounting for 47.8% (35.4kt) of emissions, followed by LDTs and HDPVs, responsible for 23.9% (17.7 kt) and 13.5% (10.0 kt) of emissions, respectively. For the other six cities, HDTs remained the largest contributor to NOx emissions, mainly because of their high EFs and annual vehicle kilometres travelled in Shenyang. HDTs were also the most important contributor of PM_10_, contributing between 48.5% and 72.5% of total PM_10_.

**Figure 6:**
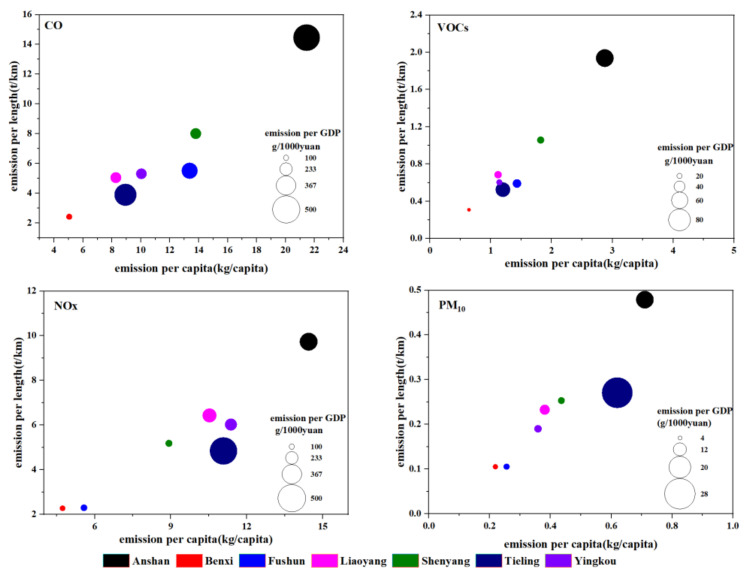
Emissions intensity, emissions per capita, and emissions per GDP of cities in the CLUA region during 2018 (GDP: gross domestic product).

Vehicle emissions are influenced by many factors and are a comprehensive reflection of city development. In the present study, we examined the relationships between pollutant emissions and road length, economic development, and population. Figure 6 shows the relationships among emission intensity (t/km), emissions per capita, and emissions per GDP for CO, NOx, VOCs, and PM_10_ at the city level. The emission intensity (t/km) in the present study was the ratio of emissions of a certain pollutant in a city to the total length of the roads. The ranges of emission intensities for each city in the CLUA region for CO, NOx, VOCs, and PM_10_ were 2.4–14.5 t/km, 2.3–9.7 t/km, 0.4–2.1 t/km, and 0.1–0.5 t/km, respectively. Notably, high emission intensity did not always equate to high emissions. For example, Shenyang had the highest emissions, yet a lower emission intensity, which was mainly due to having the longest road lengths and different road types. In contrast, Anshan and Tieling had the highest emission intensities. Anshan had the highest emissions per capita for CO, NOx, VOCs, and PM_10_, and the highest emissions per GDP for CO and VOCs, whereas Tieling had the highest emissions per GDP for NOx and PM_10_. Benxi had the lowest emission intensity, and emissions per capita and GDP. Emissions are mainly affected by vehicle ownership, the composition of vehicle types, road length, and road type in each city. 

#### 3.2.4. Spatial Distribution

**Figure 7:**
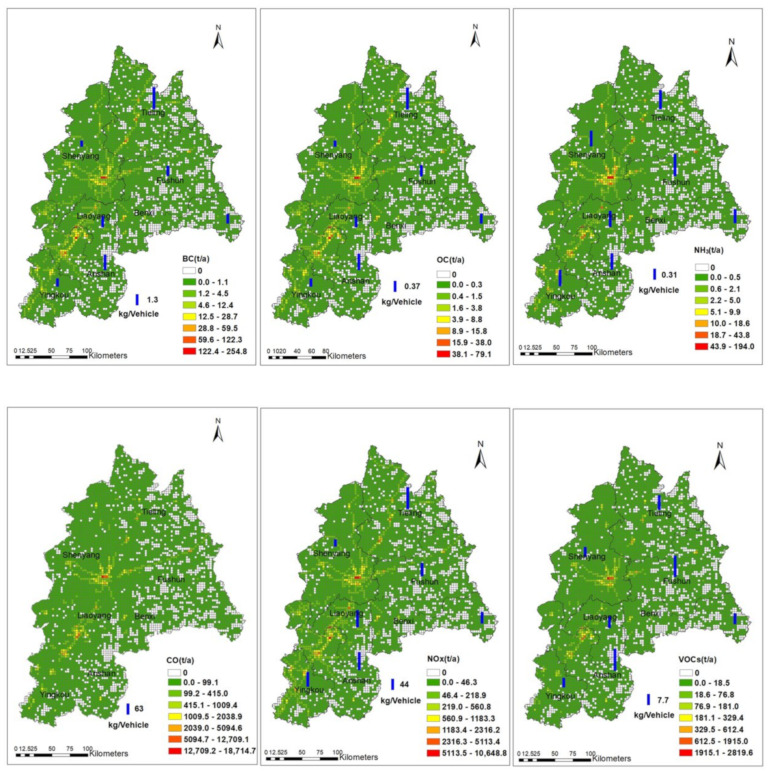

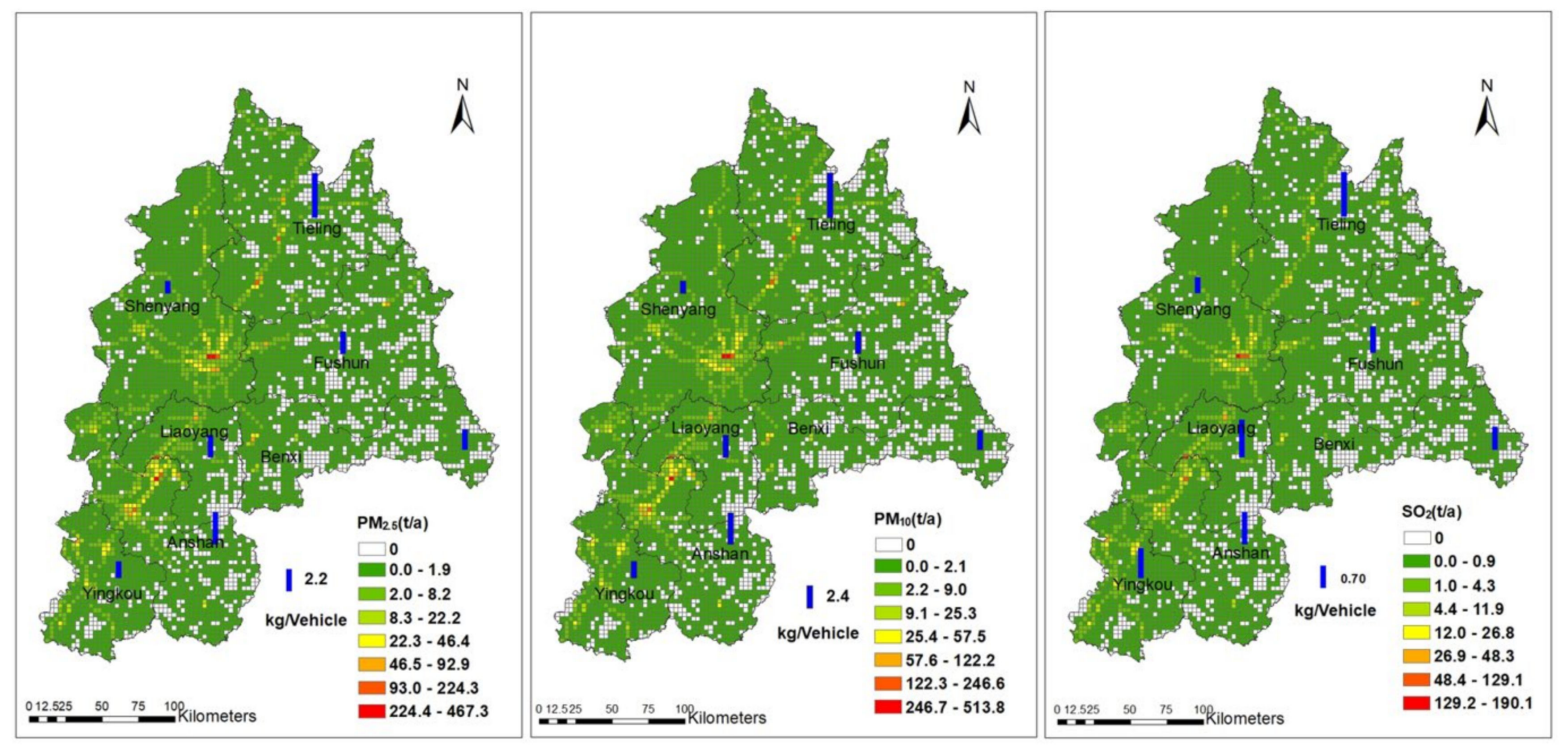
Spatial distribution of pollutant emissions and emissions per vehicle (kg/vehicle) in the CLUA region.

The spatial distribution of pollutants from vehicle emissions in the CLUA region during 2018 is illustrated in Figure 7. The size of the vertical blue column shows pollutant emissions per vehicle (kg/vehicle). For CO, emissions per vehicle in Fushun was greatest, followed by Anshan, while Shenyang had the lowest emissions per vehicle. For NOx, emissions per vehicle in Tieling was greatest, followed by Anshan and Liaoyang, with Shenyang having the lowest emissions per vehicle. For VOCs, emissions per vehicle for Anshan and Fushun were almost equal, and greater than the other cities, and emissions per vehicle for Yingkou was the smallest. For PM_10_ and PM_2.5_, Tieling had the greatest emissions per vehicle while Shenyang had the smallest. For SO_2_, NH_3_, BC, and OC, the differences in emissions per vehicle were small. Thus, the highest emissions per vehicle for NOx, SO_2_, PM_10_, PM_2.5_, BC, and OC occurred in Tieling. The reasons for this phenomenon are that the emissions for NOx, SO_2_, PM_10_, PM_2.5_, BC, and OC are mainly from heavy-duty diesel vehicles in Tieling, which accounted for 73.6%, 67.2%, 72.1%, 72.4%, 73.2%, and 71.2% of emissions, respectively. Although Shenyang had the greatest emissions, the number of motor vehicles was also significantly higher than other cities, so the average emissions per vehicle were not markedly large. 

By examining the overall spatial distribution map of pollutant emissions in the CLUA region, the emissions of various pollutants formed “obvious lines”. These “obvious lines” were consistent with the expressways, national highways, and provincial highways of the CLUA region, where higher vehicle activities and heavy traffic flows occurred. The emission characteristics of these line sources were obvious, proving that the road-network-based approach was reasonable, as shown in other cities in previous studies [8,36,44,45]. Combined with Figure S4, we find that the high pollutants emission girds are mainly in urban land use areas, while there is smaller pollutant emission distributed in rural and other construction land type grids. This is because in cities, human activities impact pollutant emission the most.

#### 3.2.5. Temporal Variation

**Figure 8:**
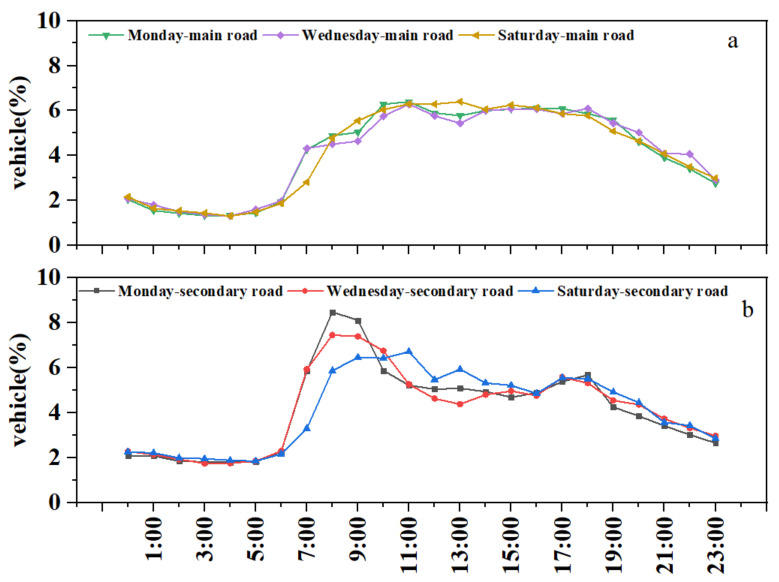
Average diurnal coefficient variations of traffic flow during the week in Shenyang for 2018 on (**a**) main and (**b**) secondary roads.

In the present study, we obtained hourly traffic flow data for first-class highways (main roads) and third-class highways (secondary roads) every Monday, Wednesday, and Saturday from the Intelligent Traffic Command Center of the Shenyang Transportation Bureau. Figure 8 shows the coefficient variation in weekly and daily traffic flow in Shenyang in January 2018. 

For main roads, the diurnal hourly variation in coefficients was higher than that at night. The hourly coefficients on weekdays and weekends were slightly different. Peaks appeared at 8:00–10:00, while troughs appeared at 13:00 on weekdays and 14:00 on weekends, which might reflect meal or lunch breaks. People travelled about an hour later on weekends than on weekdays. For secondary roads, there were obvious morning–evening peaks during the week, with the evening peak occurring at around 17:00–18:00. Differences occurred in morning peaks which appeared at 8:00 on weekdays and 11:00 on weekends. In general, hourly coefficient variations reflected people’s behaviour and schedules to some extent.

However, these variations could not reflect the true vehicle emissions due to a lack of information regarding the specific distribution of vehicles on the road in the CLUA. Thus, the traffic flow data were insufficient and future work will monitor the traffic flow of different vehicle types to improve the accuracy of the temporal vehicle emissions distribution.

### 3.3. Comparison with Other Inventories

The study results of other domestic urban agglomeration areas and the north-eastern region for vehicle emission inventories were investigated, with the differences shown in Table 4. For previous studies in the north-eastern region, the differences with the estimated results were mainly due to different years, different cities, and different methods for the handling of emission factors and vehicle populations. Yuan et al. determined the vehicle emissions of Liaoning in 2012 and found greater emissions than in this work [22]. Sergio et al. [23] combined Chinese emissions guidelines with VEIN to obtain emission factors and establish a vehicle emission inventory of NEC in 2016, which included 14 cities of Liaoning and 133 pollutants. Compared with the Harbin-Changchun Megalopolis (HCM) [46] cluster in northeast China, the number of motor vehicles and quantity of CO and VOC emissions in the CLUA region were significantly lower, and the NOx emissions were slightly higher. This is likely due to the greater contribution of HDTs in the CLUA to NOx (60.4%) compared to the HCM (41.5%). The vehicle quantity in Shenyang in the CLUA region in 2018 was larger than in 2013, and the vehicle emissions of NOx changed significantly from 2013 to 2018, increasing by 67.5%. This is attributable to the large increase in DTs (32.9%) in Shenyang from 2013 to 2018. The slight decrease in CO emissions was related to eliminating yellow-label vehicles (gasoline vehicles below China I emission standards and diesel vehicles below China III emission standards) [38] and increasing the emission standards for small passenger cars [47]. Compared with other regions, the emissions of all pollutants in the CLUA region were significantly lower than those in the Beijing-Tianjin-Hebei (BTH), Pearl River Delta (PRD), and Yangtze River Delta (YRD) areas, partly due to the small number of motor vehicles in the CLUA region.

### 3.4. Uncertainty Analysis

The uncertainty ranges of emissions for various pollutants and vehicle types in the CLUA were estimated through the Monte Carlo method and presented in Table S6. Table S7 is the sample size that was input into the Monte Carlo model. As shown in Tables S6 and S7, for vehicle fleets with a relatively small sample size, the uncertainty was relatively large. For Diesel-MPV, there were only two samples, so the uncertainty was considerably high, as shown in Table 2. The average uncertainties at the 95% confidence level were −4.3% to 4.6% for CO, −5.8% to 6.0% for NOx, −3.7% to 3.7% for VOCs, −3.5% to 3.8% for SO_2_, −9.4% to 10.5% for NH_3_, −5.8% to 6.0% for PM_10_, −5.5% to 5.9% for PM_2.5_, −5.7% to 6.4% for BC, and −5.6% to 5.9% for OC in the CLUA. For most vehicle types, the uncertainty of each pollutant emission was within ±20%. Compared with cities or other regions in northeast China, the uncertainty of this vehicle emission inventory is similar to that of a study in the HCM [46]. However, it is difficult to compare the uncertainties of previous studies [19,20,21,22] in this area as many undertook only qualitative uncertainty analysis or no uncertainty analysis.

## 4. Conclusions

In the present study, the 2018 vehicle emission inventories for CO, NOx, SO_2_, NH_3_, VOCs, PM_2.5_, PM_10_, BC, and OC at a 3 km × 3 km spatial resolution in the CLUA region were established. In 2018, vehicles emitted 291.0, 221.8, 3.6, 2.2,42.8, 9.3, 10.3, 5.2, and 1.6 Gg of CO, NOx, SO_2_, NH_3_, VOCs, PM_2.5_, PM_10_, BC, and OC, respectively. The analysis of the contributions from different vehicle types showed that diesel HDTs were the major source of NOx, SO_2_, PM_2.5_, PM_10_, BC, and OC emissions, with gasoline SPVs being the dominant contributor to CO, NH_3,_ and VOC emissions. Pre-China 1 and China 1 vehicles were the main contributors of CO and VOC emissions. The majority of the NOx, SO_2,_ and PM emissions were caused by China 3 and China 4 vehicles because 79.3% of the HDTs operated under these emission standards. The 3 km × 3 km spatial distribution of CO, NOx, SO_2_, NH_3_, VOCs, PM_2.5_, PM_10_, BC, and OC showed the highest emissions in central Shenyang, followed by Anshan. However, the emissions per vehicle (kg/vehicle) of Tieling was prominent in the CLUA. The spatial distributions of the pollutants were in agreement with the road network of the CLUA region. The vehicle flow coefficient showed morning–evening peaks, which are relevant to resident behaviour.

Therefore, this study improves our understanding of the vehicular emission characteristics of the CLUA. Additionally, this work provides a basic vehicle emission inventory for the ongoing establishment of regional air quality monitoring and the three-dimensional warning platform of the CLUA.

However, there are several limitations to the present study. Traffic flow data were obtained from only two road types in Shenyang and basic emission factors were sourced from reference data. Future research should observe local traffic flow according to different vehicle types. Additionally, a greater number of road types should be studied in each city and typical months should be selected for investigation. Furthermore, emission factors for different vehicle types should be monitored in the CLUA region. Thus, future research will help to establish a detailed vehicle emission inventory, which will provide support for the government in preparing traffic management plans in the CLUA.

## Figures and Tables

**Table 1:** Data sources used to obtain the parameters required for the CLUA vehicle inventory.

City	Statistics Level	Data Year	Data Sources
Shenyang	City	2018	Public security bureau
Yingkou	City	2017	Traffic control department and vehicle pollution supervision and management department
Liaoyang	City	2017	Supervision and management department
Benxi	City	2017	Supervision and management department
Fushun	City	2017	Supervision and management department
Anshan	Subdistrict	2017	Supervision and management department
Tieling	City	2018	Liaoning statistical yearbook

**Table 2:** Quantities of the different vehicle types and national vehicular emission limit standards in the CLUA region during 2018.

Fuel	Vehicle	Population	Emission Standard Distribution (%)					
			Pre-China 1	China 1	China 2	China 3	China 4	China 5
Gasoline	Taxi	25,951	13.6	9.1	7.6	15.8	45.2	8.8
	HDPV	6815	11.7	5.6	33.9	32.7	15.7	0.3
	Bus	581	74.5	9.3	15.9	0.2	-	-
	NM	292,709	13.6	13.3	34.2	38.8	-	0.1
	LM	35,556	77.9	11.3	6.5	3.7	-	0.5
	LDT	84,094	8.8	5.7	7.3	29.3	42.8	6.1
	MT	975	43.4	23	12.9	19.2	1.4	0.1
	MPV	54,035	12	37	16.5	18.7	15.6	0.1
	SPV	3,595,323	1.8	5.4	9.2	20.5	54	9.2
	MDT	1806	4.4	2.2	7.7	61.7	20.8	3.2
	MDPV	19,653	10.1	13.2	48.2	12	16	0.6
	HDT	4475	5.1	37.9	20.2	27.8	1.3	7.8
	subtotal	4,121,973	3.7	6.5	11.2	21.7	48.5	8.3
Diesel	Taxi	714	5.6	9.8	28.4	29.8	26.3	-
	HDPV	24,474	3	5.2	8	52.8	29.6	1.4
	LPT	30,870	16.4	18.8	64.8	-	-	-
	Bus	7733	1	8.4	6	76.8	6.4	1.4
	LDT	171,431	0.0	2	6.9	76.5	9.9	4.7
	TR	63,740	39.3	47.6	13	-	-	0.1
	MT	1008	-	-	1.1	98.9	-	-
	MPV	2	-	100	-	-	-	-
	SPV	27,033	7.1	2.9	10.7	73	3.1	3.1
	MDT	36,019	13.5	10.2	10.9	48	13	4.4
	MDPV	9556	2.5	7.8	14.4	47.4	25.5	2.4
	HDT	185,857	1.4	4.9	8.7	51.4	27.9	5.7
	subtotal	558,437	7.3	10	12	51.6	15.1	3.9
Other fuels	Taxi	28,455	0	-	0	0.6	96.7	2.7
	HDPV	8129	-	-	0.2	2.6	10.7	86.6
	Bus	6531	-	-	1.7	7.5	21.2	69.6
	LDT	210	-	-	0.5	0.5	54.3	44.8
	MPV	101	-	1	0.5	-	91.5	6.9
	SPV	35,124	0.3	0.1	0.5	1.2	84.8	13.1
	MDT	2	-	-	-	-	50	50
	MDPV	2928	-	0	2.9	1.7	68.3	27
	HDT	350	-	-	-	0.9	18	81.1
	subtotal	81,831	0.2	0	0.5	1.6	75.5	22.2
Total		4,762,241	4.1	6.8	11.1	24.9	45.1	8

HDPV: heavy-duty passenger vehicles; NM: normal motorcycles; LM: light motorcycles; LDT: light-duty trucks; MT: mini trucks; MPV: mini passenger vehicles; SPV: small passenger vehicles; MDT: medium-duty trucks; MDPV: medium-duty passenger vehicles; HDT: heavy-duty trucks; TR: Tricycle.

**Table 3:** Vehicle emissions in each city in the CLUA region in 2018 (t/a).

City	CO	NOx	SO_2_	NH_3_	VOCs	PM_2.5_	PM_10_	BC	OC
Yingkou	24,462.4	27,729.7	444.1	223.3	3211.6	792.4	875.3	438.7	131.6
Shenyang	114,394.7	74,042.1	1297.4	1158.5	17,809.7	3285.6	3615.6	1808.9	552.0
Liaoyang	15,202.4	19,338.1	328.7	129	2307.8	630.3	700.1	358.3	105.9
Anshan	77,194.2	51,959.3	738.6	347.5	11,362.7	2311.5	2556.1	1301.9	411.9
Benxi	8519	7980.9	123.2	68.7	1308.9	335.5	369.3	187.9	56.3
Fushun	27,616.8	11,511.9	182.9	137.4	3295.2	478.8	528	259.8	83.6
Tieling	23,571.5	29,228.5	468.4	181.2	3455.1	1468.9	1634.3	844.1	246.8
Total	290,960.8	221,790.6	3583.4	2245.6	42,751.0	9303	10,278.6	5199.6	1588.1

**Table 4:** Comparison of vehicle emission inventories among the CLUA and other domestic regions.

Area	Base Year	Population (10^4^)	Emission(kt/a)									Reference
			SO_2_	NOx	CO	PM_10_	PM_2.5_	VOCs	NH_3_	BC	OC	
CLUA	2018	476.2	3.6	221.8	291.0	10.3	9.3	37.5	2.2	5.2	1.6	This study
Liaoning Province	2012		4.6	288.7	789.0	14.6	13.1		2.5	7.0	2.2	[22]
BTH	2013		29.5	775.6	1853.8	47.6	46.3	176.2	4.0	26.4	9.1	[39]
PRD	2010							433.5				[48]
YRD	2010	2748.4	34.3	691.2		40.2	38.6	515.9	13.2			[12]
HCM	2016	714.0		211.3	625.6	12.2	11.2	80.5	5.2			[46]
YRD	2015	3142.0		1123.4	2943.0	47.2						[49]
PRD	2012		9.0	348.9	1859.8	35.5	28.9	213.9	4.5			[50]
NEC	2016		2.0	518.2	1420.2	19.0	17.1			10.1		[23]
Shenyang	2018	254.7	1.3	74.0	114.4	3.6	3.3	17.8	1.2	1.8	0.6	This study
Shenyang	2013	139.8		44.2	128.5	2.0						[19]

BTH: Beijing-Tianjin-Hebei; YRD: Yangtze River Delta; PRD: Pearl River Delta; HCM: Harbin-Changchun Megalopolis. NEC: Northeast China.

## Data Availability

Not applicable.

## Supplementary Materials

The following are available online at https://www.mdpi.com/article/10.3390/ijerph19042033/s1, Figure S1: The intertwined road network of CLUA region, Figure S2: Population distribution of different vehicle type in each city (**a**) and in CLUA region (**b**) in 2018, Figure S3: Emission contribution of different vehicle types of the CLUA region in 2018, Figure S4: The 3 km × 3 km grids of CLUA, Figure S5: Spatial map of traffic flow per day in CLUA, Table S1: The typical vehicle emission inventory in China [6,7,8,12,13,23,36,37,38,39,41,45,46,51,52,53,54,55,56,57,58,59,60], Table S2: The baseline emission factors of fleet types in this study, Table S3: Annual Meteorological and geographical * conditions in CLUA in 2018, Table S4: Detailed value information of other parameters, Table S5: distribution of multi-pollutant emissions of different type of vehicles in 2018, Table S6: the uncertainties range of 9 pollutants and each vehicle type in CLUA region, Table S7: Sample size of input Monte-Carlo model, Table S8. Gasoline vehicle average speed correction coefficient, Table S9. Diesel vehicle average speed correction coefficient, Table S10. Degradation correction coefficient for gasoline vehicles for 2017, Table S11. the sulfur content correction coefficient for gasoline vehicles, Table S12. Coefficient of load and oil quality.

## References

[1] National Bureau of Statistics China Statistical Yearbook 2019. http://www.stats.gov.cn/tjsj/ndsj/2019/indexch.htm.

[2] China Mobile Source Environmental Management Annual Report 2020. http://www.mee.gov.cn/xxgk2018/xxgk/xxgk15/202008/t20200810_793252.html.

[3] Tong R., Liu J., Wang W., Fang Y. (2020). Health effects of PM2.5 emissions from on-road vehicles during weekdays and weekends in Beijing, China. Atmos. Environ..

[4] Tang V., Oanh N.T.K., Rene E.R., Binh T.N. (2020). Analysis of roadside air pollutant concentrations and potential health risk of exposure in Hanoi, Vietnam. J. Environ. Sci. Health Part A Toxic Hazard. Subst. Environ. Eng..

[5] Mohammadiha A., Malakooti H., Esfahanian V. (2018). Development of reduction scenarios for criteria air pollutants emission in Tehran Traffic Sector, Iran. Sci. Total Environ..

[6] Zheng B., Huo H., Zhang Q., Yao Z.L., Wang X.T., Yang X.F., Liu H., He K.B. (2014). High-resolution mapping of vehicle emissions in China in 2008. Atmos. Chem. Phys..

[7] Liu Y.H., Liao W.Y., Li L., Huang Y.T., Xu W.J. (2017). Vehicle emission trends in China’s Guangdong Province from 1994 to 2014. Sci. Total Environ..

[8] Gong M., Yin S., Gu X., Xu Y., Jiang N., Zhang R. (2017). Refined 2013-based vehicle emission inventory and its spatial and temporal characteristics in Zhengzhou, China. Sci. Total Environ..

[9] Zhang L., Hu X., Qiu R., Lin J. (2019). Comparison of real-world emissions of LDGVs of different vehicle emission standards on both mountainous and level roads in China. Transp. Res. Part D Transp. Environ..

[10] Yang Z., Liu Y., Wu L., Martinet S., Zhang Y., Andre M., Mao H. (2020). Real-world gaseous emission characteristics of Euro 6b light-duty gasoline- and diesel-fueled vehicles. Transp. Res. Part D Transp. Environ..

[11] Huang Y., Organ B., Zhou J.L., Surawski N.C., Hong G., Chan E.F.C., Yam Y.S. (2018). Emission measurement of diesel vehicles in Hong Kong through on-road remote sensing: Performance review and identification of high-emitters. Environ. Pollut..

[12] Fu X., Wang S., Zhao B., Xing J., Cheng Z., Liu H., Hao J. (2013). Emission inventory of primary pollutants and chemical speciation in 2010 for the Yangtze River Delta region, China. Atmos. Environ..

[13] Hao J., He D., Wu Y., Fu L., He K. (2000). A study of the emission and concentration distribution of vehicular pollutants in the urban area of Beijing. Atmos. Environ..

[14] Chen W., Zhang S., Tong Q., Zhang X., Zhao H., Ma S., Xiu A., He Y. (2018). Regional Characteristics and Causes of Haze Events in Northeast China. Chin. Geogr. Sci..

[15] Sida Q. (2018). Air Pollution Characteristics and Regional Transport of PM2.5 for Liaoning Province Using Models-3/CMAQ. Master’s Thesis.

[16] Ying S. (2020). Characteristics of Air Pollution in Major Cities of Liaoning Province and Source Apportionment of PM2.5 from 2014 to 2018. Master’s Thesis.

[17] Chung J.H., Lai H., Joo J.H. (2009). Assessing the “Revive the Northeast” (zhenxing dongbei) Programme: Origins, Policies and Implementation. China Q..

[18] Liaoning Provincal Bureau of Statistics, Liaoning Statistical Yearbook 2018. http://tjj.ln.gov.cn/tjsj/sjcx/ndsj/otherpages/2018/indexch.htm.

[19] Dan W., Li-ping Z., Yan-fang Y., Guang-feng X., Xuan W., Xue Z., Wei H. (2018). Development of Vehicle Emission Inventory in Shenyang. Environ. Monit. Manag. Technol..

[20] Liping Z. (2019). Establishment and Characteristics Analysis of Air Pollution Source Emission Inventory in Benxi City. Master’s Thesis.

[21] Jiaxin J., Shida S., Peng W., Chao L.Y., Ting W., Lin W., Ning W., Junyu C., Hongjun M. (2020). Vehicle Emission Inventory and Scenario Analysis in Liaoning from 2000 to 2030. Environ. Sci..

[22] Mengchen Y., Zu B., Qingxin Z., Penghui L., Yu Z. (2018). Emission inventory and characteristics of anthropogenic air pollutant sources in Liaoning Province. Acta Sci. Circumstantiae.

[23] Ibarra-Espinosa S., Zhang X.L., Xiu A.J., Gao C.K., Wang S., Ba Q., Gao C., Chen W.W. (2021). A comprehensive spatial and temporal vehicular emissions for northeast China. Atmos. Environ..

[24] Liaoning Provincal Bureau of Statistics, Liaoning Statistical Yearbook 2020. http://tjj.ln.gov.cn/tjsj/sjcx/ndsj/otherpages/2020/2020/zk/indexch.htm.

[25] Liaoning Provincal Bureau of Statistics, Liaoning Statistical Yearbook 2017. http://tjj.ln.gov.cn/tjsj/sjcx/ndsj/otherpages/2017/indexch.htm.

[26] MEE The Announcement about Releasing Five National Technical Guidelines of the Air Pollutant Emissions Inventory. http://www.mee.gov.cn/gkml/hbb/bgg/201501/t20150107_293955.htm.

[27] Iqbal A., Afroze S., Rahman M. (2021). Probabilistic total PM2.5 emissions from vehicular sources in Australian perspective. Environ. Monit. Assess..

[28] Maes A.d.S., Hoinaski L., Meirelles T.B., Carlson R.C. (2019). A methodology for high resolution vehicular emissions inventories in metropolitan areas: Evaluating the effect of automotive technologies improvement. Transp. Res. Part D Transp. Environ..

[29] Song X., Hao Y. (2019). Vehicular Emission Inventory and Reduction Scenario Analysis in the Yangtze River Delta, China. Int. J. Environ. Res. Public Health.

[30] EMEP/EEA (2019). EMEP/EEA Air Pollutant Emission Inventory Guidebook 2019.

[31] Huo H., Zhang Q., He K., Wang Q., Yao Z., Streets D.G. (2009). High-resolution vehicular emission inventory using a link-based method: A case study of light-duty vehicles in Beijing. Environ. Sci. Technol..

[32] Hu J., Wu Y., Wang Z., Li Z., Zhou Y., Wang H., Bao X., Hao J. (2012). Real-world fuel efficiency and exhaust emissions of light-duty diesel vehicles and their correlation with road conditions. J. Environ. Sci..

[33] Liu H., Man H., Cui H., Wang Y., Deng F., Wang Y., Yang X., Xiao Q., Zhang Q., Ding Y. (2017). An updated emission inventory of vehicular VOCs and IVOCs in China. Atmos. Chem. Phys..

[34] Wu Y., Zhang S.J., Li M.L., Ge Y.S., Shu J.W., Zhou Y., Xu Y.Y., Hu J.N., Liu H., Fu L.X. (2012). The challenge to NOx emission control for heavy-duty diesel vehicles in China. Atmos. Chem. Phys..

[35] Junyu Z., Wenwei C., Zhaoli W. (2009). Traffic flow and road network-based spatial allocation of regional mobile source emission inventories. Acta Sci. Circumstantiae.

[36] Gu X., Yin S., Lu X., Zhang H., Wang L., Bai L., Wang C., Zhang R., Yuan M. (2019). Recent development of a refined multiple air pollutant emission inventory of vehicles in the Central Plains of China. J. Environ. Sci..

[37] Zhou Z., Tan Q., Liu H., Deng Y., Wu K., Lu C., Zhou X. (2019). Emission characteristics and high-resolution spatial and temporal distribution of pollutants from motor vehicles in Chengdu, China. Atmos. Pollut. Res..

[38] Liu Y.H., Ma J.L., Li L., Lin X.F., Xu W.J., Ding H. (2018). A high temporal-spatial vehicle emission inventory based on detailed hourly traffic data in a medium-sized city of China. Environ. Pollut..

[39] Qi J., Zheng B., Li M., Yu F., Chen C., Liu F., Zhou X., Yuan J., Zhang Q., He K. (2017). A high-resolution air pollutants emission inventory in 2013 for the Beijing-Tianjin-Hebei region, China. Atmos. Environ..

[40] Wang Y.J., Ji Z., Yin H., Ding Y., Su S., Qian L.Y., Wang J.F. (2014). Study of Parameters Influencing Measurement on Heavy Duty Diesel Vehicle’s Emission Factors. Res. Environ. Sci..

[41] Tao S.C., Deng S.X., Hao Y.Z., Gao S., Xiong X., Kong Y. (2019). Vehicle emission characteristics of gaseous pollutants in Guanzhong urban agglomeration. China Environ. Sci..

[42] Wu Y., Zhang S., Hao J., Liu H., Wu X., Hu J., Walsh M.P., Wallington T.J., Zhang K.M., Stevanovic S. (2017). On-road vehicle emissions and their control in China: A review and outlook. Sci. Total Environ..

[43] Guo X., Fu L., Ji M., Lang J., Chen D., Cheng S. (2016). Scenario analysis to vehicular emission reduction in Beijing-Tianjin-Hebei (BTH) region, China. Environ. Pollut..

[44] Zheng J., Che W., Wang X., Louie P., Zhong L. (2009). Road-network-Based spatial allocation of on-road mobile source emissions in the Pearl River Delta region, China, and comparisons with population-based approach. J. Air Waste Manag. Assoc..

[45] Jing B., Wu L., Mao H., Gong S., He J., Zou C., Song G., Li X., Wu Z. (2016). Development of a vehicle emission inventory with high temporal–spatial resolution based on NRT traffic data and its impact on air pollution in Beijing—Part 1: Development and evaluation of vehicle emission inventory. Atmos. Chem. Phys..

[46] Gao C., Gao C., Song K., Xing Y., Chen W. (2020). Vehicle emissions inventory in high spatial–temporal resolution and emission reduction strategy in Harbin-Changchun Megalopolis. Process Saf. Environ. Prot..

[47] Ran Z. (2017). Liaoning Province will eliminate 100,000 yellow label vehicles in 2017. China Resour. Compr. Util..

[48] Yin S., Zheng J., Lu Q., Yuan Z., Huang Z., Zhong L., Lin H. (2015). A refined 2010-based VOC emission inventory and its improvement on modeling regional ozone in the Pearl River Delta Region, China. Sci. Total Environ..

[49] Jingling W. (2018). Study and Evaluation on Pollution Characteristics and Control Policies of Motor Vehicles in the Yangtze River Delta Region. Master’s Thesis.

[50] Yang L., Zeng W., Zhang Y., Liu Y., Liao C., Gan Y., Deng X. (2015). Establishment of emission inventory and spatial-temporal allocation model for air pollutant sources in the Pearl. River Delta region. China Environ. Sci..

[51] Guo H., Zhang Q., Shi Y., Wang D. (2007). On-road remote sensing measurements and fuel-based motor vehicle emission inventory in Hangzhou, China. Atmos. Environ..

[52] Wang H., Chen C., Huang C., Fu L. (2008). On-road vehicle emission inventory and its uncertainty analysis for Shanghai, China. Sci. Total Environ..

[53] Lang J., Cheng S., Zhou Y., Zhang Y., Wang G. (2014). Air pollutant emissions from on-road vehicles in China, 1999–2011. Sci. Total Environ..

[54] Jiang P., Chen X., Li Q., Mo H., Li L. (2020). High-resolution emission inventory of gaseous and particulate pollutants in Shandong Province, eastern China. J. Clean. Prod..

[55] Jiang P., Zhong X., Li L. (2020). On-road vehicle emission inventory and its spatio-temporal variations in North China Plain. Environ. Pollut..

[56] Lv W., Hu Y., Li E., Liu H., Pan H., Ji S., Hayat T., Alsaedi A., Ahmad B. (2019). Evaluation of vehicle emission in Yunnan province from 2003 to 2015. J. Clean. Prod..

[57] Yang W., Yu C., Yuan W., Wu X., Zhang W., Wang X. (2018). High-resolution vehicle emission inventory and emission control policy scenario analysis, a case in the Beijing-Tianjin-Hebei (BTH) region, China. J. Clean. Prod..

[58] Sun S., Jin J., Xia M., Liu Y., Gao M., Zou C., Wang T., Lin Y., Wu L., Mao H. (2020). Vehicle emissions in a middle-sized city of China: Current status and future trends. Environ. Int..

[59] Zhang S., Wu Y., Liu H., Wu X., Zhou Y., Yao Z., Fu L., He K., Hao J. (2013). Historical evaluation of vehicle emission control in Guangzhou based on a multi-year emission inventory. Atmos. Environ..

[60] Liu H., Chen X., Wang Y., Han S. (2013). Vehicle Emission and Near-Road Air Quality Modeling for Shanghai, China: Based on Global Positioning System Data from Taxis and Revised MOVES Emission Inventory. Transp. Res. Rec..

