# Supplementary material for: A Comprehensive 2018-Based Vehicle Emission Inventory and Its Spatial–Temporal Characteristics in the Central Liaoning Urban Agglomeration, China
Source: Int J Environ Res Public Health. 2022 Feb 11;19(4):2033. doi: 10.3390/ijerph19042033 (PMC8872506; doi:10.3390/ijerph19042033)
Supplement: Supplementary file 1 [file ijerph-19-02033-s001.zip › ijerph-1496265-supplementary.pdf]

International Journal of
Environmental Research and Public Health

MDPI

Supplementary Materials

Table S1. A review of typical cities established vehicle emission inventory in China [1–23]

Area	Base Year	Method	Emission Pollutants	Reference
Hangzhou	2004–2005	On-road remote sensing system	CO, HC and NOx	[1]
Shanghai	2004	IVE	CO, VOC, NOx and PM	[2]
2364 counties in China	2008		CO, non-methane hydrocarbon (NMHC), NOx and $PM_{2.5}$	[3]
Beijing–Tianjin–Hebei region	2013		SO_2, NOx, $PM_{2.5}$, PM_{10}, CO, NMVOC, NH_3, BC, and OC	[4]
Chengdu	2016		CO、VOCs、NOx、SO_2、PM_{10} and NH_3	[5]
China	1999–2011	COPERT	CO, NMVOC, NOX, BC and OC	[6]
Beijing	2013		NOx, HC, CO and PM	[7]
Foshan	2014		CO, NOX, VOCs, and $PM_{2.5}$	[8]
Shandong Province	2016		$PM_{2.5}$, PM_{10}, VOCs, NOx, SO_2, and CO	[9]
53 cities within the North China Plain (NCP)	2017		BC, CO, NH_3, NMVOCs, NOx, $PM_{2.5}$, PM_{10}, and SO_2	[10]
Yunnan	2003–2015		CO, VOC, NMVOC, CH_4, NH_3, CO_2	[11]
Tianjin	2000–2030		CO, VOCs, NOx, PM10, CO_2, and SO_2	
Harbin-Changchun Megalopolis	2016	*Guideline* of MEE[1]	CO, HC, NOX, NH_3,VOCs, $PM_{2.5}$, PM_{10},	[12]

Zhengzhou	2013		SO2、NOx, HC, CO and $PM_{2.5}$, PM_{10},	[13]
Guangdong Province	1994–2014		CO, VOCs, NOx, $PM_{2.5}$, PM_{10},	[14]
Beijing–Tianjin–Hebei (BTH) region	2014		CO, HC, NOx, $PM_{2.5}$, PM_{10},	[15]
Henan Province	2015		SO2, NOx, CO, $PM_{2.5}$, PM_{10}, VOCs, VOCs-evaporation	[16]
Langfang	2011–2025		CO，NOx，VOCs，PM_{10}	[17]
Northeast China	2016		133 pollutants (CO, HC, NOX, $PM_{2.5}$, BC, CO_2 etc)	[18]
Beijing	-	MOBILE-CHINA	CO, THC, NOx	[19]
Guangzhou	2005–2009		CO, THC, NOx	[20]
Shanghai	2012	MOVES	HC, CO, NOx, $PM_{2.5}$, PM_{10},	[21]
Guanzhong urban agglomeration	2012		NOx, CO, NH_3 , SO_2, NMHC,HCHO,CH_3CHO,C_3H_4O, C_4H_6, C_6H_6,CH_4 and N_2O	[22]
Yangtze River Delta	2010	EMBEV	SO_2, NOx, $PM_{2.5}$, PM_{10}, NMVOCs and NH_3	[23]

Technical guidelines on emission inventory (GEI) from MEE (The Ministry of Ecology and Environment)

Table S2. The baseline emission factors of fleet types in this study.

Fuel	Type	Emission Standard	CO g/km	NOx g/km	SO_2 100g/km	NH_3 100g/km	VOCs 10g/km	$PM_{2.5}$ 100g/km	PM_{10} 100g/km	BC 100g/km	OC 100g/km
Gasoline	MPV	Pre-China 1	25.7	2.0	1.0	2.6	26.9	3.0	3.0	1.0	1.0
		China 1	6.7	0.4	1.0	2.6	6.6	3.0	3.0	1.0	1.0
		China 2	2.5	0.3	1.0	2.6	3.1	1.0	1.0	0.0	0.0
		China 3	1.2	0.1	1.0	2.6	1.9	1.0	1.0	0.0	0.0
		China 4	0.7	0.0	1.0	2.6	0.8	0.0	0.0	0.0	0.0
		China 5	0.5	0.0	1.0	2.6	0.6	0.0	0.0	0.0	0.0
	SPV	Pre-China 1	25.7	2.0	1.0	2.6	26.9	3.0	3.0	1.0	1.0
		China 1	6.7	0.4	1.0	2.6	6.6	3.0	3.0	1.0	1.0
		China 2	2.5	0.3	1.0	2.6	3.1	1.0	1.0	0.0	0.0
		China 3	1.2	0.1	1.0	2.6	1.9	1.0	1.0	0.0	0.0
		China 4	0.7	0.0	1.0	2.6	0.8	0.0	0.0	0.0	0.0
		China 5	0.5	0.0	1.0	2.6	0.6	0.0	0.0	0.0	0.0
	Taxi	Pre-China 1	37.0	2.2	1.0	2.6	38.4	3.0	3.0	1.0	1.0
		China 1	16.1	0.8	1.0	2.6	13.7	3.0	3.0	1.0	1.0
		China 2	7.3	0.8	1.0	2.6	9.6	1.0	1.0	0.0	0.0
		China 3	3.0	0.2	1.0	2.6	4.5	1.0	1.0	0.0	0.0
		China 4	2.5	0.1	1.0	2.6	2.8	0.0	0.0	0.0	0.0
		China 5	2.3	0.1	1.0	2.6	2.6	0.0	0.0	0.0	0.0
	MDPV	Pre-China 1	39.1	2.9	2.0	2.8	37.0	10.0	11.0	3.0	3.0
		China 1	21.4	1.8	2.0	2.8	25.7	6.0	7.0	2.0	2.0
		China 2	15.4	1.5	2.0	2.8	14.4	2.0	2.0	1.0	1.0
		China 3	4.3	0.5	2.0	2.8	3.7	1.0	1.0	0.0	0.0
		China 4	2.0	0.2	2.0	2.8	1.1	1.0	1.0	0.0	0.0
		China 5	2.0	0.2	2.0	2.8	1.1	1.0	1.0	0.0	0.0
	HDPV	Pre-China 1	100.7	5.2	2.0	2.8	51.4	29.0	33.0	8.0	9.0
		China 1	62.1	2.7	2.0	2.8	52.6	16.0	18.0	5.0	5.0
		China 2	16.6	2.6	2.0	2.8	19.8	7.0	8.0	2.0	2.0
		China 3	8.3	1.5	2.0	2.8	8.7	4.0	5.0	1.0	1.0
		China 4	3.8	0.8	2.0	2.8	4.2	4.0	5.0	1.0	1.0
		China 5	3.8	0.6	2.0	2.8	4.2	4.0	5.0	1.0	1.0
	Bus	Pre-China 1	100.7	5.2	2.0	2.8	51.4	29.0	33.0	8.0	9.0
		China 1	62.1	2.7	2.0	2.8	52.6	16.0	18.0	5.0	5.0
		China 2	16.6	2.6	2.0	2.8	19.8	7.0	8.0	2.0	2.0
		China 3	8.3	1.5	2.0	2.8	8.7	4.0	5.0	1.0	1.0
	MT	Pre-China 1	47.8	3.3	1.0	2.6	49.9	10.0	11.0	3.0	3.0
		China 1	26.2	2.0	1.0	2.6	33.2	6.0	7.0	2.0	2.0
		China 2	21.5	1.7	1.0	2.6	22.1	2.0	2.0	1.0	1.0
		China 3	5.6	0.5	1.0	2.6	6.1	1.0	1.0	0.0	0.0

		China 4	2.4	0.2	1.0	2.6	1.7	1.0	1.0	0.0	0.0
		China 5	2.4	0.2	1.0	2.6	1.7	1.0	1.0	0.0	0.0
	LDT	Pre-China 1	47.8	3.3	1.0	2.6	49.9	10.0	11.0	3.0	3.0
		China 1	26.2	2.0	1.0	2.6	33.2	6.0	7.0	2.0	2.0
		China 2	21.5	1.7	1.0	2.6	22.1	2.0	2.0	1.0	1.0
		China 3	5.6	0.5	1.0	2.6	6.1	1.0	1.0	0.0	0.0
		China 4	2.4	0.2	1.0	2.6	1.7	1.0	1.0	0.0	0.0
		China 5	2.4	0.2	1.0	2.6	1.7	1.0	1.0	0.0	0.0
	MDT	Pre-China 1	123.1	5.8	2.0	2.8	68.8	29.0	33.0	8.0	9.0
		China 1	75.8	3.0	2.0	2.8	67.8	16.0	18.0	5.0	5.0
		China 2	23.3	2.9	2.0	2.8	30.2	7.0	8.0	2.0	2.0
		China 3	10.7	1.7	2.0	2.8	13.7	4.0	5.0	1.0	1.0
		China 4	4.5	0.9	2.0	2.8	5.7	4.0	5.0	1.0	1.0
		China 5	4.5	0.7	2.0	2.8	5.7	4.0	5.0	1.0	1.0
	HDT	Pre-China 1	123.1	5.8	2.0	2.8	67.5	29.0	33.0	8.0	9.0
		China 1	75.8	3.0	2.0	2.8	67.6	16.0	18.0	5.0	5.0
		China 2	23.3	2.9	2.0	2.8	30.1	7.0	8.0	2.0	2.0
		China 3	10.7	1.7	2.0	2.8	13.5	4.0	5.0	1.0	1.0
		China 4	4.5	0.9	2.0	2.8	5.6	4.0	5.0	1.0	1.0
		China 5	4.5	0.7	2.0	2.8	5.6	4.0	5.0	1.0	1.0
	NM	Pre-China 1	14.2	0.1	0.0	0.7	20.1	3.0	3.0	1.0	1.0
		China 1	9.0	0.1	0.0	0.7	9.9	2.0	2.0	1.0	1.0
		China 2	2.6	0.2	0.0	0.7	5.3	1.0	1.0	0.0	0.0
		China 3	1.1	0.1	0.0	0.7	2.1	0.0	0.0	0.0	0.0
		China 5	0.9	0.1	0.0	0.6	1.7	0.0	0.0	0.0	0.0
	LM	Pre-China 1	9.6	0.1	0.0	0.7	54.0	3.0	3.0	1.0	1.0
		China 1	4.2	0.1	0.0	0.7	21.5	2.0	2.0	1.0	1.0
		China 2	2.0	0.1	0.0	0.7	16.5	1.0	1.0	0.0	0.0
		China 3	0.8	0.1	0.0	0.7	8.8	0.0	0.0	0.0	0.0
		China 5	0.7	0.1	0.0	0.6	7.0	0.0	0.0	0.0	0.0
Diesel	MPV	China 1	0.4	1.0	5.0	0.4	0.8	6.0	7.0	4.0	1.0
	SPV	Pre-China 1	1.3	1.3	5.0	0.4	8.9	18.0	20.0	10.0	3.0
		China 1	0.4	1.0	5.0	0.4	0.8	6.0	7.0	4.0	1.0
		China 2	0.5	1.0	5.0	0.4	0.5	5.0	6.0	3.0	1.0
		China 3	0.1	0.8	5.0	0.4	0.3	3.0	4.0	2.0	1.0
		China 4	0.1	0.7	5.0	0.4	0.2	3.0	3.0	2.0	1.0
		China 5	0.1	0.7	5.0	0.4	0.2	3.0	3.0	2.0	1.0
	Taxi	Pre-China 1	1.3	1.3	5.0	0.4	8.9	18.0	20.0	10.0	3.0
		China 1	0.4	1.0	5.0	0.4	0.8	6.0	7.0	4.0	1.0
		China 2	0.5	1.0	5.0	0.4	0.5	5.0	6.0	3.0	1.0
		China 3	0.1	0.8	5.0	0.4	0.3	3.0	4.0	2.0	1.0
		China 4	0.1	0.7	5.0	0.4	0.2	3.0	3.0	2.0	1.0

MDPV	Pre-China 1	3.9	5.5	14.0	1.7	23.6	160.0	178.0	91.0	29.0
	China 1	3.4	4.8	14.0	1.7	22.5	46.0	52.0	26.0	8.0
	China 2	2.8	5.7	14.0	1.7	6.7	16.0	17.0	9.0	3.0
	China 3	2.1	3.4	14.0	1.7	5.8	15.0	16.0	8.0	3.0
	China 4	1.8	2.7	14.0	1.7	5.8	11.0	12.0	6.0	2.0
	China 5	1.8	2.3	14.0	1.7	5.8	5.0	6.0	3.0	1.0
HDPV	Pre-China 1	10.5	12.4	14.0	1.7	42.2	129.0	143.0	73.0	23.0
	China 1	9.9	11.2	14.0	1.7	9.1	98.0	109.0	56.0	18.0
	China 2	8.7	9.9	14.0	1.7	5.5	88.0	98.0	50.0	16.0
	China 3	6.7	9.9	14.0	1.7	4.5	40.0	44.0	23.0	7.0
	China 4	3.3	9.9	14.0	1.7	1.7	25.0	28.0	14.0	5.0
	China 5	1.6	8.6	14.0	1.7	0.9	13.0	14.0	7.0	2.0
Bus	Pre-China 1	10.5	12.4	14.0	1.7	42.2	129.0	143.0	73.0	23.0
	China 1	9.9	11.2	14.0	1.7	9.1	98.0	109.0	56.0	18.0
	China 2	8.7	9.9	14.0	1.7	5.5	88.0	98.0	50.0	16.0
	China 3	6.7	9.9	14.0	1.7	4.5	40.0	44.0	23.0	7.0
	China 4	3.3	9.9	14.0	1.7	1.7	25.0	28.0	14.0	5.0
	China 5	1.6	8.6	14.0	1.7	0.9	13.0	14.0	7.0	2.0
MT	China 2	3.2	5.6	5.0	0.4	14.8	26.0	29.0	15.0	5.0
	China 3	1.9	3.8	5.0	0.4	4.2	13.0	14.0	7.0	2.0
LDT	Pre-China 1	3.3	6.8	5.0	0.4	23.7	44.0	48.0	25.0	8.0
	China 1	4.2	5.6	5.0	0.4	23.1	27.0	30.0	15.0	5.0
	China 2	3.2	5.6	5.0	0.4	14.8	26.0	29.0	15.0	5.0
	China 3	1.9	3.8	5.0	0.4	4.2	13.0	14.0	7.0	2.0
	China 4	1.5	2.6	5.0	0.4	2.1	6.0	6.0	3.0	1.0
	China 5	1.5	2.2	5.0	0.4	2.1	1.0	1.0	1.0	0.0
MDT	Pre-China 1	12.1	10.8	14.0	1.7	56.2	132.0	145.0	75.0	24.0
	China 1	4.2	7.5	14.0	1.7	25.5	91.0	101.0	52.0	16.0
	China 2	4.6	6.2	14.0	1.7	6.7	27.0	30.0	16.0	5.0
	China 3	2.1	6.2	14.0	1.7	3.2	17.0	19.0	10.0	3.0
	China 4	1.7	4.4	14.0	1.7	1.6	10.0	11.0	6.0	2.0
	China 5	1.7	3.7	14.0	1.7	1.6	2.0	2.0	1.0	0.0
HDT	Pre-China 1	13.6	13.8	14.0	1.7	64.5	132.0	145.0	75.0	24.0
	China 1	5.8	9.6	14.0	1.7	14.2	62.0	69.0	36.0	11.0
	China 2	3.1	7.9	14.0	1.7	8.2	50.0	56.0	29.0	9.0
	China 3	2.8	7.9	14.0	1.7	4.0	24.0	27.0	14.0	4.0
	China 4	2.2	5.6	14.0	1.7	2.0	14.0	15.0	8.0	2.0
	China 5	2.2	4.7	14.0	1.7	2.0	3.0	3.0	2.0	0.0
LPT	Pre-China 1	4.5	4.0	5.0	0.4	14.9	18.0	19.0	10.0	3.0
	China 1	2.6	3.9	5.0	0.4	13.1	16.0	17.0	9.0	3.0
	China 2	2.1	3.1	5.0	0.4	8.5	12.0	13.0	7.0	2.0

	TR	Pre-China 1	2.0	1.1	5.0	0.4	4.5	7.0	8.0	4.0	1.0
		China 1	1.0	1.1	5.0	0.4	2.7	6.0	7.0	4.0	1.0
		China 2	0.8	0.9	5.0	0.4	1.8	5.0	5.0	3.0	1.0
		China 5	0.6	0.7	4.0	0.3	1.4	4.0	4.0	2.4	0.8
Other fuels	MPV	China 1	2.2	0.5	0.0	0.0	2.4	3.0	3.0	1.0	1.0
		China 2	1.3	0.1	0.0	0.0	1.6	1.0	1.0	0.0	0.0
		China 4	0.5	0.0	0.0	0.0	0.6	0.0	0.0	0.0	0.0
		China 5	0.5	0.0	0.0	0.0	0.9	0.0	0.0	0.0	0.0
	SPV	Pre-China 1	17.5	1.7	0.0	0.0	22.4	3.0	3.0	1.0	1.0
		China 1	2.2	0.5	0.0	0.0	2.4	3.0	3.0	1.0	1.0
		China 2	1.3	0.1	0.0	0.0	1.6	1.0	1.0	0.0	0.0
		China 3	0.8	0.1	0.0	0.0	0.9	1.0	1.0	0.0	0.0
		China 4	0.5	0.0	0.0	0.0	0.6	0.0	0.0	0.0	0.0
		China 5	0.5	0.0	0.0	0.0	0.9	0.0	0.0	0.0	0.0
	Taxi	Pre-China 1	28.8	1.8	0.0	0.0	37.9	3.0	3.0	1.0	1.0
		China 2	3.0	0.2	0.0	0.0	4.0	1.0	1.0	0.0	0.0
		China 3	0.8	0.1	0.0	0.0	1.2	1.0	1.0	0.0	0.0
		China 4	0.5	0.0	0.0	0.0	0.7	0.0	0.0	0.0	0.0
		China 5	2.3	0.0	0.0	0.0	2.9	0.0	0.0	0.0	0.0
	MDPV	Pre-China 1	9.1	6.0	0.0	0.0	19.2	10.0	11.0	3.0	3.0
		China 1	7.6	4.8	0.0	0.0	16.0	6.0	7.0	2.0	2.0
		China 2	6.1	4.0	0.0	0.0	14.3	2.0	2.0	1.0	1.0
		China 3	3.2	2.6	0.0	0.0	8.6	1.0	1.0	0.0	0.0
		China 4	2.3	1.8	0.0	0.0	6.0	1.0	1.0	0.0	0.0
		China 5	2.3	1.1	0.0	0.0	6.0	1.0	1.0	0.0	0.0
	HDPV	China 2	12.1	13.1	0.0	0.0	28.6	7.0	8.0	2.0	2.0
		China 3	6.4	9.3	0.0	0.0	17.2	4.0	5.0	1.0	1.0
		China 4	4.7	6.5	0.0	0.0	11.9	4.0	5.0	1.0	1.0
		China 5	4.6	3.7	0.0	0.0	11.9	4.0	5.0	1.0	1.0
	Bus	China 2	12.1	13.1	0.0	0.0	28.6	7.0	8.0	2.0	2.0
		China 3	6.4	9.3	0.0	0.0	17.2	4.0	5.0	1.0	1.0
		China 4	4.7	6.5	0.0	0.0	11.9	4.0	5.0	1.0	1.0
		China 5	4.6	3.7	0.0	0.0	11.9	4.0	5.0	1.0	1.0
	LDT	China 2	1.3	0.1	0.0	0.0	1.6	1.0	1.0	0.0	0.0
		China 3	0.8	0.1	0.0	0.0	0.9	1.0	1.0	0.0	0.0
		China 4	0.5	0.0	0.0	0.0	0.6	0.0	0.0	0.0	0.0
		China 5	0.5	0.0	0.0	0.0	0.9	0.0	0.0	0.0	0.0
	MDT	China 4	2.3	1.8	0.0	0.0	6.0	1.0	1.0	0.0	0.0
		China 5	2.3	1.1	0.0	0.0	6.0	1.0	1.0	0.0	0.0
	HDT	China 3	6.4	9.3	0.0	0.0	17.2	4.0	5.0	1.0	1.0
		China 4	4.7	6.5	0.0	0.0	11.9	4.0	5.0	1.0	1.0
		China 5	4.7	6.5	0.0	0.0	11.9	4.0	5.0	1.0	1.0

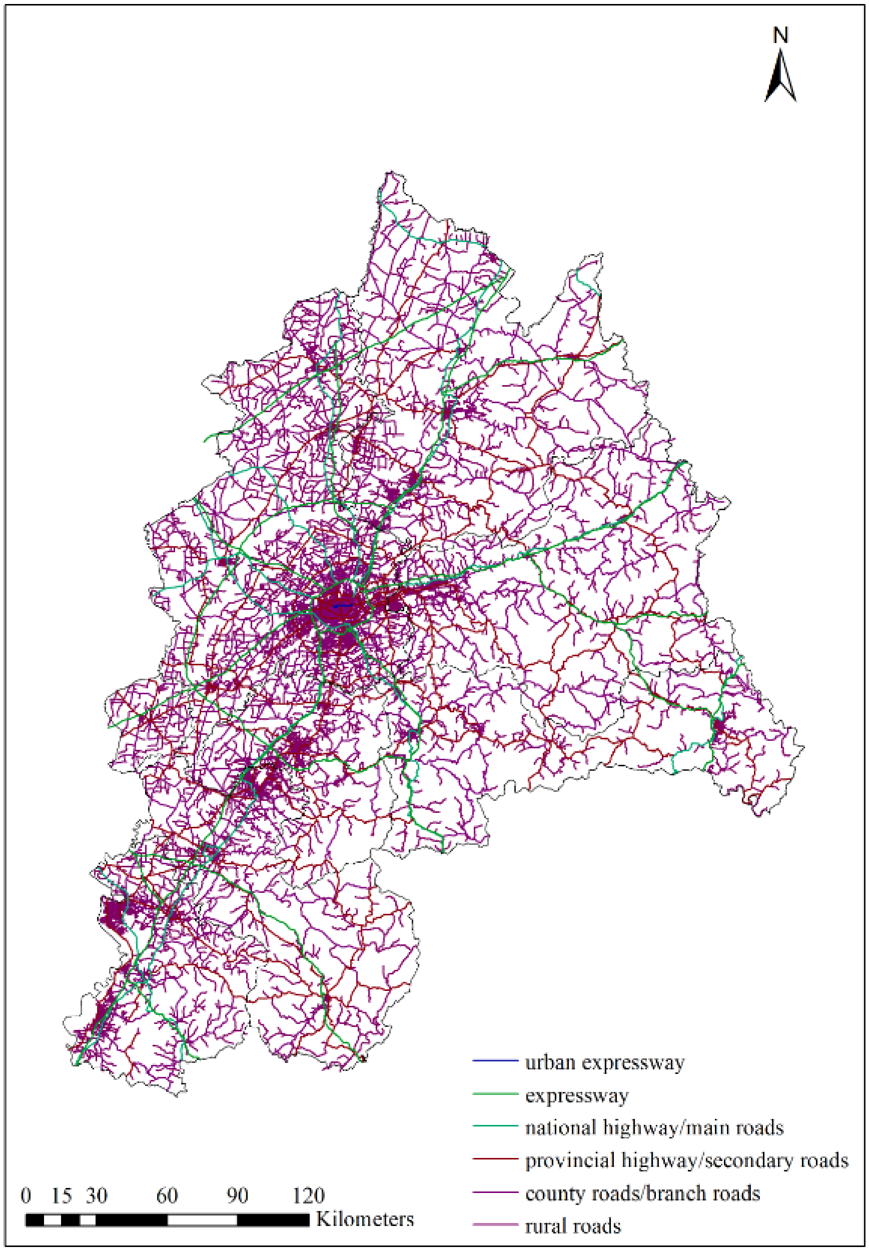

Figure S1. The intertwined road network of CLUA region.

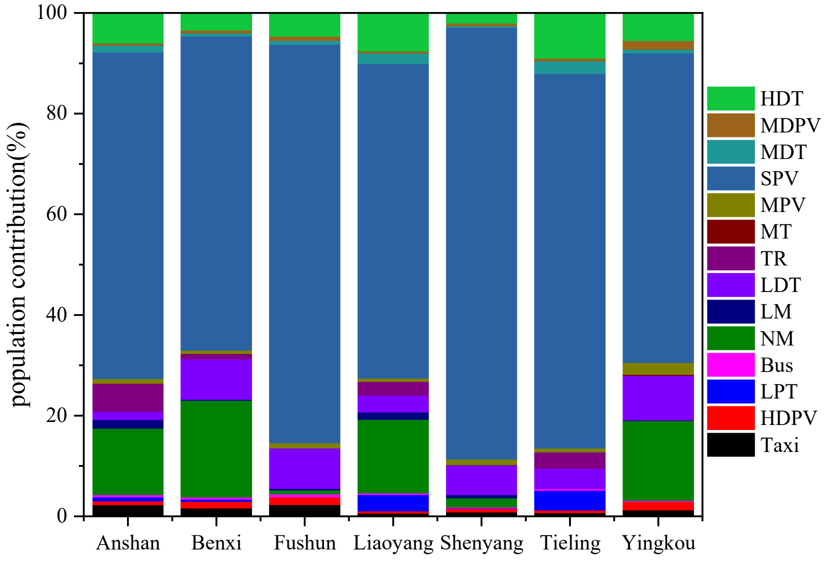

(**a**)

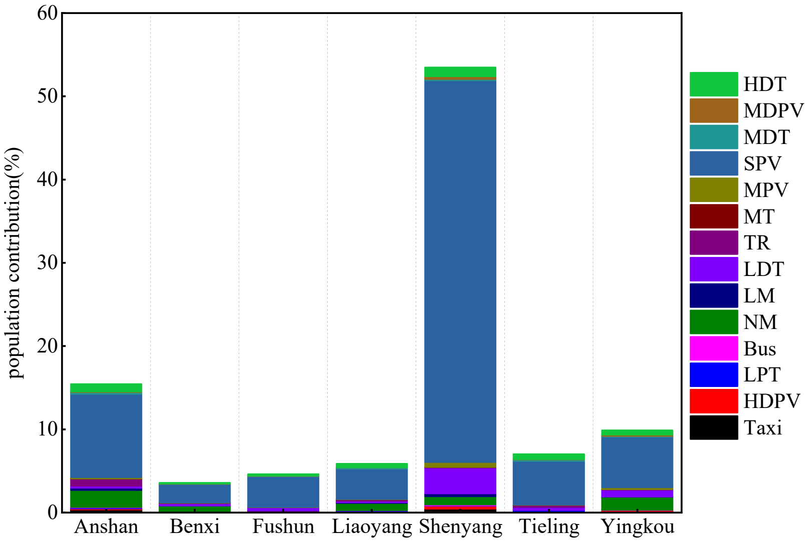

(**b**)

Figure S2. Population distribution of different vehicle type in each city (**a**) and in CLUA region (**b**) in 2018.

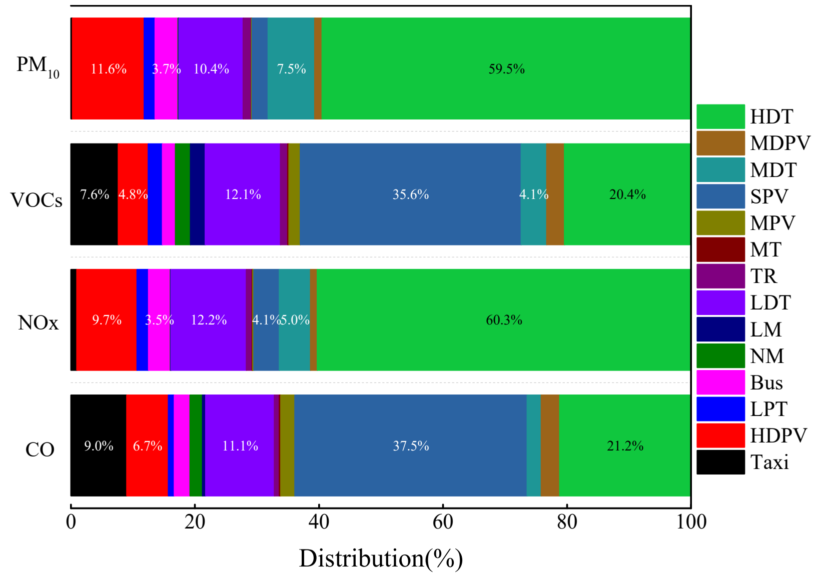

Figure S3. Emission contribution of different vehicle types of the CLUA region in 2018. The contribution less than 3% is not marked.

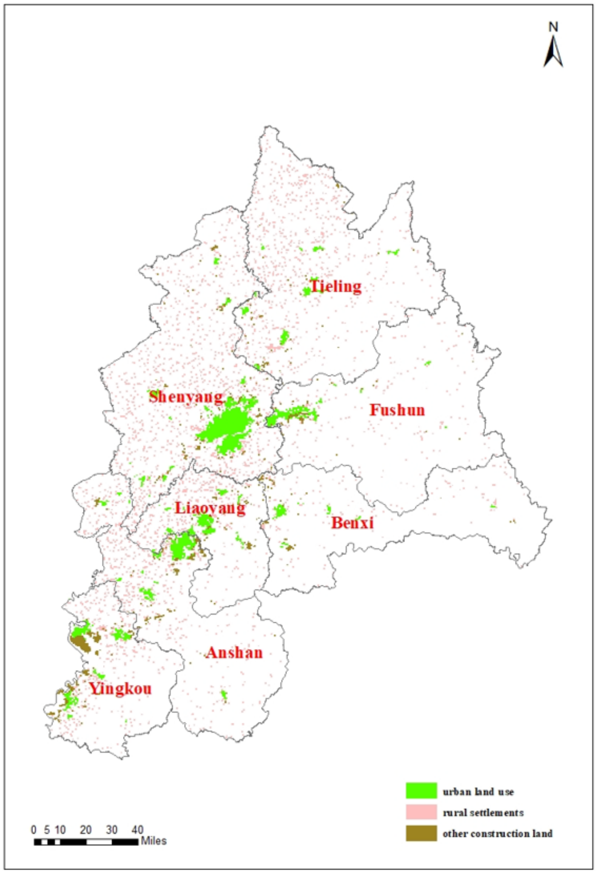

Figure S4. Map of land use types of CLUA. "**urban land use**" refers to land used in large, medium and small cities and built-up areas above counties and towns; "**rural settlements**" refers to rural settlements separated from towns and cities. "**other construction land**" refers to factories and mines, large industrial areas, oil fields, salt farms, quarries and other land, as well as roads, airports and special land.

Table S3. Annual meteorological and geographical * conditions in CLUA in 2018.

City	Average temperature (℃)	Average relative humidity (%)
Shenyang	9.3	58.8
Anshan	11.6	47.3
Fushun	7.7	63.8
Benxi	9.2	57.3
Yingkou	10.8	61.3
Liaoyang	10.5	55.3

Tieling [a]	7.9	62

*: seven cities of CLUA are in the Northeast of China, which should be classified as "low altitudes" in GEI.

[a]: data of average temperature and relative humidity of Tieling are from website. Data of other six cities are from the Statistical Yearbook of Liaoning Province.

Table S4. Detailed value information of other parameters

Factor Type	Fuel Type	Reference Standards	Value Source	Value
BEF	Gasoline	2017 as the basic year	Appendix F3 of GEI *	Table S2
	Diesel			
	Other fuel			
γ	Gasoline	30~40km/h	Appendix F9 of GEI	Table S8
	Diesel		Appendix F10 of GEI	Table S9
	Other fuel	\	\	1
λ	Gasoline	2017 as the basic year	Appendix F11 of GEI	Table S10
	Diesel	\	\	1
	Other fuel	\	\	1
Sulfur content correction coefficient	Gasoline	10ppm	Appendix F12 of GEI	Table S11
	Diesel	350ppm	Appendix F13 of GEI	1
	Other fuel	\	\	1
Load coefficient	Gasoline	\	\	1
	Diesel	100% loaded	Appendix F15 of GEI	Table S12
	Other fuel	\	\	1
Gasoline ethanol blending degree	Gasoline	10%	Appendix F14 of GEI	Table S12
	Diesel	\	\	1
	Other fuel	\	\	1

* GEI: the technical guidelines on emission inventory (GEI) and released by Ministry of Ecology and Environment of the People's Republic of China (MEE)

Table S5. distribution of multi-pollutant emissions of different type of vehicles in 2018

Fuel	Vehicle	Emission Distribution of Different Types of Vehicles (%)								
		CO	NOx	SO_2	NH_3	VOCs	$PM_{2.5}$	PM_{10}	BC	OC
Gasoline	Taxi	8.2	0.8	0.9	3.6	6.6	0.2	0.2	0.1	0.3
	HDPV	2.4	0.4	0.2	0.5	1.4	0.2	0.2	0.1	0.4
	Bus	0.7	0.1	0.0	0.0	0.3	0.1	0.1	0.0	0.1
	NM	2.0	0.1	-	0.5	4.0	0.1	0.1	0.1	0.2
	LM	0.5	0.0	-	0.1	2.4	0.0	0.0	0.0	0.1
	LDT	7.0	0.9	0.7	2.9	5.7	0.4	0.4	0.1	0.5
	MT	0.2	0.0	0.0	0.0	0.2	0.0	0.0	0.0	0.0
	MPV	2.2	0.2	0.3	1.1	1.9	0.1	0.1	0.1	0.2
	SPV	37.3	3.8	18.1	75.3	39.5	2.3	2.1	0.6	2.0
	MDT	0.3	0.0	0.0	0.1	0.2	0.0	0.0	0.0	0.0
	MDPV	2.6	0.3	0.3	0.8	2.0	0.1	0.1	0.1	0.3
	HDT	4.4	0.4	0.2	0.4	3.0	0.2	0.2	0.1	0.4
	subtotal	67.9	7.1	20.8	85.4	67.2	3.9	3.6	1.3	4.4
Diesel	Taxi	0.0	0.0	0.1	0.0	0.0	0.1	0.1	0.1	0.1
	HDPV	3.5	8.4	5.5	1.1	1.5	11.1	11.1	11.3	11.8
	LPT	1.0	1.9	1.3	0.2	2.0	1.8	1.8	1.9	1.8
	Bus	1.2	2.7	1.7	0.3	0.5	3.4	3.4	3.5	3.6
	LDT	4.1	11.3	7.2	0.9	5.4	10.2	10.0	10.0	9.6
	TR	0.8	0.9	2.0	0.3	1.0	1.2	1.3	1.4	1.2
	MT	0.0	0.1	0.0	0.0	0.0	0.1	0.0	0.0	0.0
	MPV	0.0	0.0	0.0	0.0	0.0	0.0	0.0	0.0	0.0
	SPV	0.1	0.3	0.7	0.1	0.1	0.4	0.5	0.5	0.6
	MDT	2.0	5.0	4.9	1.0	3.4	7.5	7.5	7.7	7.9
	MDPV	0.3	0.7	1.2	0.2	0.5	1.1	1.1	1.1	1.2
	HDT	16.8	59.9	54.5	10.6	14.9	58.8	59.2	61.1	57.2
	subtotal	29.8	91.0	79.2	14.6	29.4	95.7	95.9	98.5	95.0
Other fuels	Taxi	0.8	0.1	-	-	0.7	0.0	0.0	0.0	0.0

	HDPV	0.8	0.9	-	-	1.3	0.2	0.2	0.1	0.3
	Bus	0.6	0.8	-	-	1.1	0.2	0.2	0.1	0.2
	LDT	0.0	0.0	-	-	0.0	0.0	0.0	-	-
	MPV	0.0	0.0	-	-	0.0	0.0	0.0	0.0	0.0
	SPV	0.1	0.0	-	-	0.1	0.0	0.0	0.0	0.0
	MDT	0.0	0.0	-	-	0.0	0.0	0.0	-	-
	MDPV	0.1	0.1	-	-	0.1	0.0	0.0	0.0	0.0
	HDT	0.0	0.1	-	-	0.1	0.0	0.0	0.0	0.0
	subtotal	2.3	1.9	-	-	3.4	0.4	0.4	0.2	0.5
Total		100	100	100	100	100	100	100	100	100

Table S6. The uncertainty range *of nine pollutants and each vehicle type in CLUA region (unit %)

Fuel	Vehicle	CO	NOx	SO_2	NH_3	VOCs	$PM_{2.5}$	PM_{10}	BC	OC
Gasoline	Taxi	(−11.69~11.76)	(−8.9~9.52)	(−8.8~9.33)	(−8.73~9.48)	(−8.03~8.45)	(−9.88~10.63)	(−9.92~10.88)	(−12.79~13.93)	(−12.49~14.03)
	HDPV	(−10.1~10.58)	(−10.94~12.35)	(−11.68~13)	(−11.6~13.06)	(−9.32~10.09)	(−10~10.36)	(−9.86~10.57)	(−9.64~10.42)	(−9.97~10.75)
	Bus	(−21~25)	(−34~35)	(0~0)	(0~0)	(−19~21)	(0~0)	(0~0)	(0~0)	(0~0)
	NM	(−0.17~0.18)	(0~0)	-	(0~0)	(−0.06~0.06)	(0~0)	(0~0)	(0~0)	(0~0)
	LM	(−25.62~28.33)	(0~0)	-	(0~0)	(−18.23~21.58)	(0.06~0.06)	(0.06~0.06)	(0.2~0.2)	(0.2~0.2)
	LDT	(−11.07~11.25)	(0~0)	(0.01~0.01)	(0~0)	(−7.45~7.73)	(−0.01~−0.01)	(0.01~0.01)	(−0.02~−0.02)	(−0.02~−0.02)
	MT	(−10.75~11.66)	(−27~27.01)	(−3.45~3.45)	(−3.95~3.95)	(−9.49~10.4)	(−3.45~3.45)	(−3.13~3.13)	(0~0)	(0~0)
	MPV	(−15.22~17.35)	(−14.36~16.65)	(−9.85~10.81)	(−9.85~11.14)	(−13.06~14.2)	(−13.13~14.3)	(−13.2~14.41)	(−15.75~17.04)	(−15.81~17.6)
	SPV	(−9.72~10.43)	(−9.06~9.82)	(−12.51~13.93)	(−12.46~13.96)	(−7.69~8.12)	(−10.12~10.66)	(−9.85~11.05)	(−13.42~14.69)	(−13.35~14.46)
	MDT	(−12.23~13.59)	(−14.58~16.4)	(−16.18~17.82)	(−16.36~18.11)	(−12.34~13.61)	(−12.65~14.25)	(−12.96~14.83)	(−12.84~13.98)	(−12.15~13.88)
	MDPV	(−11.98~13.2)	(−12.26~13.43)	(−11.08~11.59)	(−10.93~11.95)	(−11.49~12.36)	(−11.01~11.76)	(−11.08~11.85)	(−12.5~13.2)	(−12.24~13.46)
	HDT	(−21.07~24.59)	(−15.04~17.03)	(−13.81~15.66)	(−13.85~16.03)	(0~0)	(−17.37~20.32)	(−17.42~20.02)	(−18.23~21.45)	(−18.54~21.23)
Diesel	Taxi	(−100~7.55)	(−9.6~10.18)	(−10.77~12.1)	(−12.32~13.13)	(−10.77~11.76)	(−11.16~12.27)	(−10.23~11.18)	(−11.26~12.5)	(−13.15~14.84)
	HDPV	(−14.76~15.75)	(−12.41~13.5)	(−11.5~12.65)	(−11.3~12.75)	(−9.79~10.68)	(−11.34~12.89)	(−11.37~12.65)	(−11.32~12.72)	(−11.29~12.51)
	Bus	(−13.4~13.51)	(−11.43~12.05)	(−10.44~11.14)	(−10.33~11)	(−9.65~10.44)	(−10.41~11.22)	(−10.17~11.21)	(−10.53~11.39)	(−10.18~11.22)
	LDT	(−17.19~19.2)	(−17.89~20.59)	(−17.67~19.7)	(−17.5~20.31)	(−16.47~18.45)	(−18.12~21.23)	(−18.66~21.06)	(−18.34~20.92)	(0~0)

	TR	(−25.34~26.05)	(−27.5~27.63)	(0~0)	(0~0)	(0~0)	(0~0)	(0~0)	(0~0)	(0~0)
	MT	(−29.74~34.66)	(−29.46~35.63)	(−29.35~34.69)	(−25.76~31.99)	(−29.41~34.13)	(−28.92~34.11)	(−28.98~34.79)	(−29.04~34.46)	(−27.7~35.06)
	LPT	(−12.48~13.53)	(−13.59~15.24)	(−14.33~16.04)	(−14.92~15.87)	(−12.45~13.91)	(−13.56~15.08)	(−13.73~15.58)	(−13.72~15.47)	(−13.49~14.73)
	MPV	(−34.86~30.28)	(−35.98~28.03)	(−100~−100)	(−100~−100)	(−100~−100)	(−100~111.04)	(−100~80.97)	(−100~−100)	(−100~−100)
	SPV	(−13.9~15.58)	(−16.39~19.13)	(−17.08~19.94)	(−16.75~19.74)	(−18.86~21.93)	(−15.5~17.84)	(−16.47~18.85)	(−16.27~18.54)	(−18.02~20.92)
	MDT	(−8.07~8.84)	(−7.71~8.34)	(−7.87~8.47)	(−7.96~8.43)	(−9.82~10.9)	(−8.29~8.71)	(−8.11~8.65)	(−8.18~8.76)	(−8.08~8.98)
	MDPV	(−9.31~10.32)	(−9.34~10.12)	(−10.23~11.12)	(−10.04~10.82)	(−8.46~8.9)	(−9.47~10.16)	(−9.25~9.83)	(−9.31~10)	(−9.56~10.26)
	HDT	(−7.84~8.45)	(−8.64~9.39)	(−8.73~9.35)	(−8.66~9.36)	(−6.98~7.4)	(−8.7~9.13)	(−8.58~9.09)	(−8.57~9.19)	(0~0)
Other	Taxi	(−20.78~24.34)	(−22.99~27.05)	-	-	(−20.61~24.53)	(−26.11~31.36)	(−25.85~27.71)	(−40.66~18.69)	(−40.54~18.92)
	HDPV	(−19.59~23.23)	(−18.16~21.12)	-	-	(−19.69~23.07)	(−19.94~23.11)	(−20.39~23.17)	(−20.4~23.64)	(−20.1~23.44)
	Bus	(−0.28~0.29)	(−0.27~0.27)	-	-	(0~0)	(0~0)	(0~0)	(0~0)	(0~0)
	LDT	(−22.17~24.59)	(−21.74~21.74)	-	-	(−22.81~24.35)	(−100~100)	(−100~100)	(−100~100)	(−100~100)
	MPV	(−17.49~20.6)	(−12.52~24.97)	-	-	(−14.34~19.93)	(−45.39~59.14)	(−49.43~70.11)	(−56.65~77.26)	(−53.74~77.52)
	SPV	(−21.85~26.16)	(−21.64~26)	-	-	(−20.49~25.16)	(−13.36~13.72)	(−13.35~13.73)	(−21.08~18.38)	(−21.07~18.39)
	MDT	(−20.25~28.83)	(−19.78~30.36)	-	-	(−28.44~19.26)	(−41.05~60.47)	(−45.95~61.66)	-	-
	MDPV	(−16.12~17.69)	(−16.67~19.17)	-	-	(−15.74~18.01)	(−16.22~17.71)	(−16.34~17.55)	(−26.19~10.71)	(−26.32~10.52)
	HDT	(−26~31.11)	(−24~27.98)	-	-	(−26.17~31.11)	(−26.63~30.55)	(−26.71~30.55)	(−27.51~29.72)	(−27.58~29.59)

*: at a 95% confidence level

Table S7. Sample size of input Monte-Carlo model.

Fuel	Vehicle	CO	NOx	SO_2	NH_3	VOCs	$PM_{2.5}$	PM_{10}	BC	OC
Gasoline	Taxi	50	50	50	50	100	33	33	13	13
	HDPV	62	62	62	62	124	62	62	62	62
	Bus	19	19	19	19	19	19	19	19	19
	NM	47	47		47	94	34	34	22	22
	LM	41	41		41	82	30	30	22	22
	LDT	71	71	71	71	142	71	71	37	37
	MT	44	44	44	44	88	44	44	30	30
	MPV	67	67	67	67	134	50	50	24	24
	SPV	76	76	76	76	152	50	50	24	24
	MDT	28	28	28	28	56	28	28	28	28
	MDPV	47	47	47	47	94	47	47	27	27
	HDT	48	48	48	48	96	48	48	48	48
Diesel	Taxi	30	30	30	30	30	30	30	30	30
	HDPV	63	63	63	63	63	63	63	63	63
	Bus	44	44	44	44	44	44	44	44	44
	LDT	36	36	36	36	36	36	36	26	26

	TR	34	34	34	34	34	34	34	34	34
	MT	5	5	5	5	5	5	5	5	5
	LPT	32	32	32	32	32	32	32	32	32
	MPV	1	1	1	1	1	1	1	1	1
	SPV	53	53	53	53	53	53	53	53	53
	MDT	64	64	64	64	64	64	64	64	59
	MDPV	54	54	54	54	54	54	54	54	54
	HDT	64	64	64	64	64	64	64	64	59
	Taxi	28	28			28	7	7	3	3
	HDPV	25	25			25	25	25	25	25
	Bus	21	21			21	21	21	21	21
	LDT	4	4			4	2	2		
Other	MPV	13	13			13	3	3	1	1
	SPV	51	51			51	27	27	7	7
	MDT	2	2			2	2	2		
	MDPV	23	23			23	23	23	7	7
	HDT	3	3			3	3	3	3	3

Table S8. Gasoline vehicle average speed correction coefficient.

Pollutants	Speed Range 30–40(km/h)
CO	0.79
VOCs	0.78
NOx	0.9
$PM_{2.5}$、PM_{10}、BC、OC	0.78

Table S9. Diesel vehicle average speed correction coefficient.

Pollutants	Emission Standards	Speed Range 30–40(km/h)
CO	Pre-China 1 - China 3	0.89
	China 4 - China	0.93
VOCs	Pre-China 1 - China 3	0.9
	China 4 - China	0.91
NOx	Pre-China 1 - China 3	0.93
	China 4 - China	0.91
$PM_{2.5}$、PM_{10}、BC、OC	Pre-China 1 - China 3	0.93
	China 4 - China	0.91

Table S10. Degradation correction coefficient for gasoline vehicles for 2017.

Pollutants	Vehicle Type	Pre-China 1	China 1	China 2	China 3	China 4-China 5
CO	Mini/small GVs	1.25	1.73	1.03	1.34	1.01
	Other vehicle type	1.17	1.15	1.05	1.17	1.31
	Taxi	1.27	1.57	1.59	1.46	1.62
VOCs	Mini/small GVs	1.21	1.38	1.53	1.18	1.01
	Other vehicle type	1.17	1.08	1.09	1.16	1.34
	Taxi	1.24	1.45	1.58	1.39	1.52
NOx	Mini/small GVs	1.04	1	1.32	1.47	1
	Other vehicle type	1.04	1.06	1.11	1.1	1.18
	Taxi	1.06	1.41	1.51	1.36	1.67

$PM_{2.5}$、PM_{10}、BC、OC	Mini/small GVs	1	1	1	1	1
	Other vehicle type	1	1	1	1	1
	Taxi	1	1	1	1	1

Table S11. The sulfur content correction coefficient for gasoline vehicles.

Emission Standard\Pollutant	CO	VOCs	NOx	$PM_{2.5}$、PM_{10}、BC、OC
Pre-China 1	0.9	0.96	0.95	1
China 1	0.9	0.96	0.95	1
China 2	0.9	0.96	0.95	1
China 3	0.9	0.96	0.95	1
China 4	0.9	0.96	0.95	1
China 5	0.9	0.96	0.95	1

Table S12. Coefficient of load and oil quality.

**Type**	**CO**	**VOCs**	**NOx**	**$PM_{2.5}$、PM_{10}、BC、OC**
load coefficient	1.33	1	1.43	1.26
Gasoline ethanol blending degree	0.84	0.82	1	0.82

Note: The sulfur content correction coefficient for diesel vehicles for all pollutants is 1. The correction coefficient for pollutants not mentioned here (Table S8-S12) have a default value of 1.

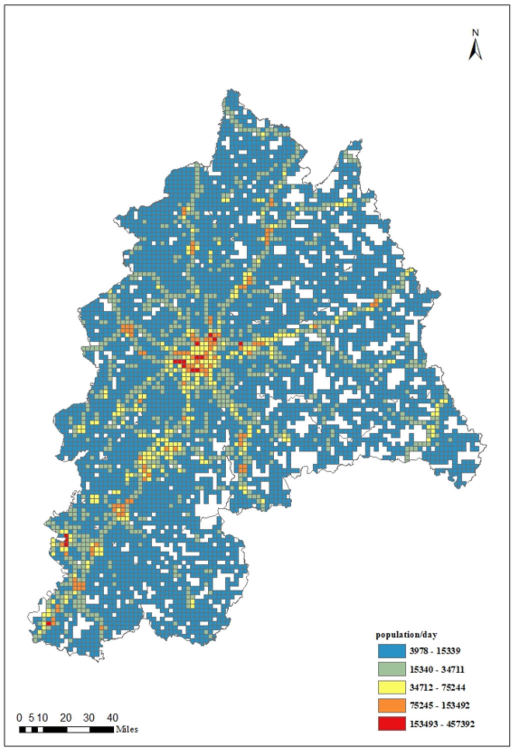

Figure S5. Spatial map of traffic flow per day in CLUA.

Figure S5: Traffic flow of different road types of each city, distributed in each grid. Note that the number of vehicles per grid in each city is the sum of all vehicle types in a different road pattern in each grid.

References:

1. Guo, H.; Zhang, Q.; Shi, Y.; Wang, D. On-road remote sensing measurements and fuel-based motor vehicle emission inventory in Hangzhou, China. *Atmos. Environ.* **2007**, *41*, 3095–3107, https://doi.org/10.1016/j.atmosenv.2006.11.045.
2. Wang, H.; Chen, C.; Huang, C.; Fu, L. On-road vehicle emission inventory and its uncertainty analysis for Shanghai, China. *Sci Total. Env.* **2008**, *398*, 60–67, https://doi.org/10.1016/j.scitotenv.2008.01.038.
3. Zheng, B.; Huo, H.; Zhang, Q.; Yao, Z.L.; Wang, X.T.; Yang, X.F.; Liu, H.; He, K.B. High-resolution mapping of vehicle emissions in China in 2008. *Atmos. Chem. Phys.* **2014**, *14*, 9787–9805. https://doi.org/10.5194/acp-14-9787-2014.
4. Qi, J.; Zheng, B.; Li, M.; Yu, F.; Chen, C.; Liu, F.; Zhou, X.; Yuan, J.; Zhang, Q.; He, K. A high-resolution air pollutants emission inventory in 2013 for the Beijing-Tianjin-Hebei region, China. *Atmos. Environ.* **2017**, *170*, 156–168. https://doi.org/10.1016/j.atmosenv.2017.09.039.
5. Zhou, Z.; Tan, Q.; Liu, H.; Deng, Y.; Wu, K.; Lu, C.; Zhou, X. Emission characteristics and high-resolution spatial and temporal distribution of pollutants from motor vehicles in Chengdu, China. *Atmos. Pollut. Res.* **2019**, *10*, 749–758. https://doi.org/10.1016/j.apr.2018.12.002.
6. Lang, J.; Cheng, S.; Zhou, Y.; Zhang, Y.; Wang, G. Air pollutant emissions from on-road vehicles in China, 1999–2011. *Sci. Total. Environ.* **2014**, *496*, 1–10, https://doi.org/10.1016/j.scitotenv.2014.07.021.
7. Jing, B.; Wu, L.; Mao, H.; Gong, S.; He, J.; Zou, C.; Song, G.; Li, X.; Wu, Z. Development of a vehicle emission inventory with high temporal–spatial resolution based on NRT traffic data and its impact on air pollution in Beijing—Part 1: Development and evaluation of vehicle emission inventory. *Atmos. Chem. Phys.* **2016**, *16*, 3161–3170. https://doi.org/10.5194/acp-16-3161-2016.
8. Liu, Y.H.; Ma, J.L.; Li, L.; Lin, X.F.; Xu, W.J.; Ding, H. A high temporal-spatial vehicle emission inventory based on detailed hourly traffic data in a medium-sized city of China. *Environ. Pollut.* **2018**, *236*, 324–333. https://doi.org/10.1016/j.envpol.2018.01.068.
9. Jiang, P.; Chen, X.; Li, Q.; Mo, H.; Li, L. High-resolution emission inventory of gaseous and particulate pollutants in Shandong Province, eastern China. *J. Clean. Prod.* **2020**, *259*, 120806, https://doi.org/10.1016/j.jclepro.2020.120806.
10. Jiang, P.; Zhong, X.; Li, L. On-road vehicle emission inventory and its spatio-temporal variations in North China Plain. *Environ. Pollut.* **2020**, *267*, 115639, https://doi.org/10.1016/j.envpol.2020.115639.
11. Lv, W.; Hu, Y.; Li, E.; Liu, H.; Pan, H.; Ji, S.; Hayat, T.; Alsaedi, A.; Ahmad, B. Evaluation of vehicle emission in Yunnan province from 2003 to 2015. *J. Clean. Prod.* **2019**, *207*, 814–825, https://doi.org/10.1016/j.jclepro.2018.09.227.
12. Gao, C.; Gao, C.; Song, K.; Xing, Y.; Chen, W. Vehicle emissions inventory in high spatial–temporal resolution and emission reduction strategy in Harbin-Changchun Megalopolis. *Process Saf. Environ. Prot.* **2020**, *138*, 236–245. https://doi.org/10.1016/j.psep.2020.03.027.
13. Gong, M.; Yin, S.; Gu, X.; Xu, Y.; Jiang, N.; Zhang, R. Refined 2013-based vehicle emission inventory and its spatial and temporal characteristics in Zhengzhou, China. *Sci. Total Environ.* **2017**, *599-600*, 1149–1159. https://doi.org/10.1016/j.scitotenv.2017.03.299.
14. Liu, Y.H.; Liao, W.Y.; Li, L.; Huang, Y.T.; Xu, W.J. Vehicle emission trends in China's Guangdong Province from 1994 to 2014. *Sci. Total Environ.* **2017**, *586*, 512–521. https://doi.org/10.1016/j.scitotenv.2017.01.215.
15. Yang, W.; Yu, C.; Yuan, W.; Wu, X.; Zhang, W.; Wang, X. High-resolution vehicle emission inventory and emission control policy scenario analysis, a case in the Beijing-Tianjin-Hebei (BTH) region, China. *J. Clean. Prod.* **2018**, *203*, 530–539, https://doi.org/10.1016/j.jclepro.2018.08.256.
16. Gu, X.; Yin, S.; Lu, X.; Zhang, H.; Wang, L.; Bai, L.; Wang, C.; Zhang, R.; Yuan, M. Recent development of a refined multiple air pollutant emission inventory of vehicles in the Central Plains of China. *J. Environ. Sci.* **2019**, *84*, 80–96. https://doi.org/10.1016/j.jes.2019.04.010.

17. Sun, S.; Jin, J.; Xia, M.; Liu, Y.; Gao, M.; Zou, C.; Wang, T.; Lin, Y.; Wu, L.; Mao, H.; et al. Vehicle emissions in a middle-sized city of China: Current status and future trends. *Environ. Int.* **2020**, *137*, 105514, https://doi.org/10.1016/j.envint.2020.105514.
18. Ibarra-Espinosa, S.; Zhang, X.L.; Xiu, A.J.; Gao, C.K.; Wang, S.; Ba, Q.; Gao, C.; Chen, W.W. A comprehensive spatial and temporal vehicular emissions for northeast China. *Atmos. Environ.* **2021**, *244*, 12. https://doi.org/10.1016/j.atmosenv.2020.117952.
19. Hao, J.; He, D.; Wu, Y.; Fu, L.; He, K. A study of the emission and concentration distribution of vehicular pollutants in the urban area of Beijing. *Atmos. Environ.* **2000**, *34*, 453–465.
20. Zhang, S.; Wu, Y.; Liu, H.; Wu, X.; Zhou, Y.; Yao, Z.; Fu, L.; He, K.; Hao, J. Historical evaluation of vehicle emission control in Guangzhou based on a multi-year emission inventory. *Atmos. Environ.* **2013**, *76*, 32–42, https://doi.org/10.1016/j.atmosenv.2012.11.047.
21. Liu, H.; Chen, X.; Wang, Y.; Han, S. Vehicle Emission and Near-Road Air Quality Modeling for Shanghai, China: Based on Global Positioning System Data from Taxis and Revised MOVES Emission Inventory. *Transp. Res. Rec.* **2013**, *2340*, 38–48, https://doi.org/doi:10.3141/2340-05.
22. Tao, S.C.; Deng, S.X.; Hao, Y.Z.; Gao, S.; Xiong, X.; Kong, Y. Vehicle emission characteristics of gaseous pollutants in Guanzhong urban agglomeration. *China Environ. Sci.* **2019**, *39*, 542–553. https://doi.org/10.19674/j.cnki.issn1000-6923.2019.0065.
23. Fu, X.; Wang, S.; Zhao, B.; Xing, J.; Cheng, Z.; Liu, H.; Hao, J. Emission inventory of primary pollutants and chemical speciation in 2010 for the Yangtze River Delta region, China. *Atmos. Environ.* **2013**, *70*, 39–50. https://doi.org/10.1016/j.atmosenv.2012.12.034.
